# Impact of the SARS-CoV-2 pandemic on hematopoietic cell transplantation and cellular therapies in Europe 2020: a report from the EBMT activity survey

**DOI:** 10.1038/s41409-022-01604-x

**Published:** 2022-02-22

**Authors:** Jakob R. Passweg, Helen Baldomero, Christian Chabannon, Selim Corbacioglu, Rafael de la Cámara, Harry Dolstra, Bertram Glass, Raffaella Greco, Mohamad Mohty, Bénédicte Neven, Régis Peffault de Latour, Zinaida Perić, John A. Snowden, Ibrahim Yakoub-Agha, Anna Sureda, Nicolaus Kröger, for the European Society for Blood and Marrow Transplantation (EBMT)

**Affiliations:** 1 EBMT Activity Survey Office, Hematology Division, University Hospital, Basel, Switzerland; 2 Institut Paoli Calmettes Comprehensive Cancer Center and Inserm CBT-1409, Centre d’Investigations Cliniques en Biothérapies, Marseille, France; 3 Department of Pediatric Hematology, Oncology and Stem Cell Transplantation, University of Regensburg, Regensburg, Germany; 4 Hematology Department, Hospital Universitario Sanitas La Zarzuela, Madrid, Spain; 5 Laboratory of Hematology, Department of Laboratory Medicine, Radboud University Medical Center, Nijmegen, The Netherlands; 6 Klinik für Hämatologie und Stammzelltransplantation, HELIOS Klinikum Berlin-Buch, Berlin, Germany; 7 Unit of Hematology and Bone Marrow Transplantation, IRCCS San Raffaele Scientific Institute, Vita-Salute San Raffaele University, Milan, Italy; 8 Department of Hematology, Hospital Saint Antoine, Sorbonne University, INSERM UMRs938, Paris, France; 9 Pediatric Immune-Hematology Unit, Necker Children Hospital, Assistance Publique Hôpitaux de Paris, Paris, France; 10 BMT Unit, Department of Hematology, Hospital St. Louis, Paris, France; 11 Hematology Department, University Hospital Center Rebro, Zagreb, Croatia; 12 Department of Haematology, Sheffield Teaching Hospitals NHS Foundation Trust, Sheffield, UK; 13 CHU de Lille, Université de Lille, INSERM U1286, Infinite, Lille, France; 14 Clinical Hematology Department, Institut Català d’Oncologia-Hospitalet, Institut d’Investigació Biomèdica de Bellvitge (IDIBELL), University of Barcelona, Barcelona, Spain; 15 Department of Stem Cell Transplantation, University Hospital Eppendorf, Hamburg, Germany

**Keywords:** Haematological cancer, Leukaemia

## Abstract

In 2020, 45,364 HCT in 41,016 patients, 18,796 (41%) allogeneic and 26,568 (59%) autologous in 690 centers were reported. Changes observed were as follows: total number of HCT −6.5%, allogeneic HCT −5.1%, autologous HCT −7.5%, and were more pronounced in non-malignant disorders for allogeneic HCT and in autoimmune disease for autologous HCT. Main indications were myeloid malignancies 10,441 (25%), lymphoid malignancies 26,120 (64%) and non-malignant disorders 2532 (6%). A continued growth in CAR-T cellular therapies to 1874 (+65%) patients in 2020 was observed. In allogeneic HCT, the use of haploidentical donors increased while use of unrelated and sibling donors decreased. Cord blood HCT increased by 11.7% for the first time since 2012. There was a significant increase in the use of non-myeloablative but a drop in myeloablative conditioning and in use of marrow as stem cell source. We interpreted these changes as being due to the SARS-CoV-2 pandemic starting early in 2020 in Europe and provided additional data reflecting the varying impact of the pandemic across selected countries and larger cities. The transplant community confronted with the pandemic challenge, continued in providing patients access to treatment. This annual report of the EBMT reflects current activities useful for health care planning.

## Introduction

The European Society for Blood and Marrow Transplantation (EBMT) published first in 1990 [1] a survey describing activity in hematopoietic stem cell transplant centers in Europe that was published annually thereafter. The survey spanning 31 years includes over 750,000 patients with over 850,000 transplants. Initially the survey was designed in the form of a single page spreadsheet for ease of reporting and has remained in this format ever since. Many additional features have been added, such as refined disease classification, information on conditioning intensity, pediatric activity and stem cell source. Most recently hematopoietic cellular therapies that qualify as medicinal products rather than cell transplants have been included.

Hematopoietic cell transplantation (HCT) is an established procedure for many acquired or inherited disorders of the hematopoietic system, benign or neoplastic, including those of the immune system, and as enzyme replacement in metabolic disorders [2–4]. The activity survey of the EBMT, describing the status of HCT, has become an instrument with which to observe trends and monitor changes in HCT technology in Europe and neighboring countries [5–15]. The survey, using a standardized structure, captured the numbers of HCT from highly committed participating centers, stratified by indication, donor type and stem cell source over time [16, 17, https://www.ema.europa.eu/en/documents/scientific-guideline/qualification-opinion-cellular-therapy-module-european-society-blood-marrow-transplantation-ebmt_en.pdf]. In the last few years, the survey also included information on cellular therapies with hematopoietic cells for uses other than to replace the hematopoietic system [18–27]. The analysis of the survey data since 1990 has illustrated a continued and constant increase in the annual numbers of HCT and transplant rates for both allogeneic and autologous HCT [14]. This 2020 survey data report, showed for the first time in 31 years a drop in activity possibly related to the SARS-CoV-2 pandemic [28–32].

## Patients and methods

### Data collection and validation

We invited participating centers to report their data for 2020 using the activity survey as shown in Table 1. Patients who received their first transplant in the survey year are reported in the main table by disease, donor type and stem cell source. Additional information on the numbers of subsequent transplants performed due to relapse, rejection, or those that are part of a planned sequential protocol are reported as a summary below the main table. Information on the numbers of patients receiving un-manipulated donor lymphocyte infusions (DLIs), non-myeloablative or reduced intensity HCT, and the numbers of pediatric HCT were also collected.

**Table 1:** Numbers of HCT in Europe 2020 by indication, donor type and stem cell source.

	Transplant activity 2020																	
	No. of patients																	
	Allogeneic												Autologous			Total		
	Family									Unrelated						Allo	Auto	Total
	HLA-id			Twin	Haplo ≥ 2MM		Other family						BM	BM+				
	BM	PBPC	Cord	all	BM	PBPC	BM	PBPC	Cord	BM	PBPC	cord	only	PBPC	cord			
Myeloid malignancies	245	2413	9	8	291	1569	12	84	5	253	5209	119	0	224	0	10,217	224	10,441
Acute myeloid leukemia	167	1697	0	6	229	1117	9	67	4	143	3324	94	0	222	0	6857	222	7079
1st complete remission	118	1124	0	3	131	583	6	36	3	94	1862	53	0	186	0	4013	186	4199
Not 1st complete remission	38	393	0	3	65	374	3	24	1	36	861	30	0	31	0	1828	31	1859
AML therapy-related or myelodysplasia-related changes	11	180	0	0	33	160	0	7	0	13	601	11	0	5	0	1016	5	1021
Chronic myeloid leukemia	9	106	1	0	7	40	0	4	0	15	171	1	0	0	0	354	0	354
Chronic phase	4	56	1	0	5	17	0	3	0	9	67	1	0	0	0	163	0	163
Not chronic phase	5	50	0	0	2	23	0	1	0	6	104	0	0	0	0	191	0	191
MDS or MD/MPN overlap	62	432	7	1	46	308	3	9	1	87	1231	23	0	0	0	2210	0	2210
MPN	7	178	1	1	9	104	0	4	0	8	483	1	0	2	0	796	2	798
Lymphoid malignancies	280	1252	4	11	199	924	9	48	3	200	2038	78	32	21,042	0	5046	21,074	26,120
Acute lymphatic leukemia	237	720	4	4	139	537	7	31	3	173	1106	63	0	54	0	3024	54	3078
1st complete remission	132	498	3	1	68	274	3	16	3	81	739	31	0	51	0	1849	51	1900
Not 1st complete remission	105	222	1	3	71	263	4	15	0	92	367	32	0	3	0	1175	3	1178
Chronic lymphocytic leukemia	2	42	0	0	2	17	0	2	0	2	98	4	0	33	0	169	33	202
Plasma cell disorders—MM	1	88	0	5	8	21	0	1	0	1	134	0	11	12,354	0	259	12,365	12,624
Plasma cell disorders—other	0	7	0	0	1	4	0	0	0	1	17	0	0	401	0	30	401	431
Hodgkin lymphoma	12	100	0	0	17	129	1	3	0	2	108	3	9	2125	0	375	2134	2509
Non-Hodgkin lymphoma	28	295	0	2	32	216	1	11	0	21	575	8	12	6075	0	1189	6087	7276
Solid tumors	1	0	0	0	7	24	2	0	0	0	2	0	18	1667	1	36	1686	1722
Neuroblastoma	0	0	0	0	7	20	1	0	0	0	0	0	12	492	1	28	505	533
Soft tissue sarcoma/Ewing	1	0	0	0	0	3	0	0	0	0	0	0	3	278	0	4	281	285
Germinal tumors	0	0	0	0	0	0	0	0	0	0	0	0	1	455	0	0	456	456
Other solid tumors	0	0	0	0	0	1	1	0	0	0	2	0	2	442	0	4	444	448
Non-malignant disorders	586	267	21	4	119	207	58	47	2	370	469	63	4	315	0	2213	319	2532
Bone marrow failure—SAA	168	122	0	4	35	55	8	6	0	118	151	9	0	1	0	676	1	677
Bone marrow failure—other	43	21	1	0	14	22	10	6	0	51	53	5	0	0	0	226	0	226
Thalassemia	120	27	12	0	3	9	12	6	0	33	53	0	0	6	0	275	6	281
Sickle cell disease	107	56	5	0	18	11	5	1	0	15	6	0	0	1	0	224	1	225
Primary immune deficiencies	124	27	3	0	43	92	18	25	0	107	160	22	4	1	0	621	5	626
Inh. disorders of Metabolism	23	10	0	0	6	18	5	2	2	42	38	27	0	8	0	173	8	181
Autoimmune disease—MS	1	0	0	0	0	0	0	0	0	0	0	0	0	226	0	1	226	227
Autoimmune disease—SSC	0	1	0	0	0	0	0	0	0	0	0	0	0	49	0	1	49	50
Autoimmune disease—other	0	3	0	0	0	0	0	1	0	4	8	0	0	23	0	16	23	39
Others	28	15	0	0	5	17	2	5	0	19	37	7	0	66	0	135	66	201
Total patients	1140	3947	34	23	621	2741	83	184	10	842	7755	267	54	23,314	1	17,647	23,369	41,016
Re/additional transplants	29	123	2	1	46	382	4	11	1	46	473	31	3	3196	0	1149	3199	4348
Total transplants	1169	4070	36	24	667	3123	87	195	11	888	8228	298	57	26,510	1	18,796	26,568	45,364

In addition in Table 2, centers reported information on different types of cellular therapies qualifying as advanced therapy medicinal products (ATMP). These therapies result from substantial manipulations of collected cells, whether manufactured by industry centrally or locally by an academic institution.

**Table 2:** Numbers of patients treated with a cellular therapy in Europe 2020 by indication, donor type and cell source.

Number of patients	DLI	CART		MSC		NK cells		Selected/expanded T cells or CIK		Regulatory T cells (TREGS)		Genetically modified T cells		Dendritic cells		Expanded CD34+ cells		Genetically modified CD34+ cells		Other		Total excl DLI	
2020		Allo	Auto	Allo	Auto	Allo	Auto	Allo	Auto	Allo	Auto	Allo	Auto	Allo	Auto	Allo	Auto	Allo	Auto	Allo	Auto	allo	auto
GvHD				343	1					24				1				1				369	1
Graft enhancement				31	2	6		13	5							13		1		159	35	223	42
Autoimmune dis.				22	9																	22	9
Genetic disease												1							8			1	8
Infection				17				147				2						1		21	1	188	1
Malignancy—ALL		25	291	1		2		41							4					3		72	295
Malignancy—HL/NHL		2	1435			1		7				4								2	1	16	1436
Malignancy—Other		2	120			14		7	36	5			5	5	26				5	8	4	41	196
DLI for graft enhancement/failure	728																					0	0
DLI for residual disease	482																					0	0
DLI for relapse	1265																					0	0
DLI per protocol	580																					0	0
Total	3055	29	1846	414	12	23	0	215	41	29	0	7	5	6	30	13	0	3	13	193	41	932	1988

Quality control measures included several independent systems: confirmation of validity of the entered data by the reporting center, selective comparison of the survey data with MED-A data sets in the EBMT Registry database and crosschecking with National Registries.

### Participating centers

Since 1990, a directory of HCT centers consisting of both members of the EBMT and non-members, in both European and collaborating non-European countries has been accrued. The directory is updated annually according to the centers current activity. In 2020, 720 centers from 53 countries were contacted (44 European and 9 collaborating countries); of which 690 centers responded. This corresponded to a 96% return rate and included 17% EBMT non-members. Twenty-nine active centers failed to report in 2020. Reporting centers are listed in the Supplementary online Appendix in alphabetical order, by country, city, and EBMT center code, with their reported numbers of first and total HCT, and of first allogeneic and autologous HCT. The WHO regional office definitions were used to classify countries as European or non-European. Nine collaborating non-European countries participated in the 2020 survey: Algeria, Iran, Iraq, Jordan, Lebanon, Nigeria, Saudi Arabia, South Africa, and Tunisia. Their data, 2328 HCT in 2252 patients, from 27 actively transplanting centers made up 5.1% of the total data set and were included in all analyses.

### Patient and transplant numbers

Wherever appropriate, patient numbers corresponding to the number of patients receiving a first transplant in 2020, and transplant numbers reflecting the total number of transplants performed were listed. The term sibling donor included HLA identical siblings and twins but not siblings with HLA mismatches. Unrelated donor transplants included HCT from matched or mismatched unrelated donors with peripheral blood and bone marrow as a stem cell source but not cord blood HCT. Haploidentical transplants were described as any family member with a full haplotype mismatch. Other family member donors were those related donors that are mismatched to a lesser degree than a full haplotype. For the purpose of analysis we added the small number of “other family donor” to haploidentical donor HCT. Additional non-first transplants included multiple transplants defined as subsequent transplants within a planned double or triple autologous or allogeneic transplant protocol, and re-transplants (autologous or allogeneic) defined as unplanned HCT for rejection, poor-graft function or relapse after a previous HCT. To specifically analyze the impact of the SARS-CoV-2 pandemic on transplantation technology we restricted a sub-analysis for specific countries and transplant centers in some major cities to center’s with complete reported data for the years 2017–2020 (*N* = 653 centers) in order to compare annual changes in transplant numbers.

### Hematopoietic advanced cellular therapies other than hematopoietic cell transplantation

Centers were requested to report all patients receiving cellular therapies other than HCT in 2020. Hematopoietic advanced cellular therapies were defined as infusion of cells undergoing substantial manipulation after collection, either selection and/or expansion, or genetic modification and thus qualify as investigational or approved ATMPs according to Regulation (EC) No. 1394/2007. In this context, “substantial” should be understood as referring to the definition included in the Regulation and subsequent regulatory documents and may not reflect the workload assumed by cell processing facilities working in conjunction with clinical programs. Depending on their nature and indications, hematopoietic cellular therapies may be designed to replace or to complement HCT. Administration of non-substantially manipulated hematopoietic cells, such as transplantation of CD34+ selected hematopoietic stem cells were counted as HCT and not as cellular therapy [18]. Similarly, un-manipulated lymphocyte infusions post-HCT were counted as DLI and not cellular therapy. Hematopoietic cellular therapies include immune effector cells as defined in FACT-JACIE standards for Hematopoietic Cellular Therapy: “A cell that has differentiated into a form capable of modulating or effecting a specific immune response” [17, 33]. This definition covered CAR-T cells and formed the basis for accreditation requirements in recent EBMT-JACIE recommendations [33].

Hematopoietic cellular therapies were categorized as chimeric antigen receptor T cells (CAR-T); in vitro selected/and or expanded T cells or cytokine activated, such as virus specific T cells; cytokine-induced killer cells; regulatory T cells (TREGS); genetically modified T cells other than CAR-T; natural killer cells; dendritic cells; mesenchymal stromal cells; in vitro expanded CD34+ cells; and genetically modified CD34+ cells. This survey did not include cells from sources other than hematopoietic tissue. On the other hand, gene therapy protocols, such as those used to treat thalassemia or SCID were included, however numbers have remained low.

### Transplant and cellular therapy rates

Transplant rate, defined as the total number of HCT per 10 million inhabitants were computed for each country (based on the centers reports), without adjusting for patients receiving their HCT in a foreign country. Cellular therapy rates were defined as the numbers of patients receiving a cellular therapy treatment per 10 million population. Population numbers for the European countries in 2020 were obtained from Eurostats: (https://ec.europa.eu/eurostat) and the World Bank database for the non-European countries: (https://databank.worldbank.org).

### Analysis

Wherever appropriate, the absolute numbers of transplanted patients, transplants or transplant rates were shown for specific countries, indications, or transplant techniques. To study impact of the SARS-CoV-2 pandemic in specific countries and transplant centers in major cities, we compared mean values of change in absolute transplant numbers from 2017–2018, 2018–2019 and 2019–2020, depicting these graphically. Myeloid malignancy included acute myeloid leukemia (AML), myelodysplastic or myelodysplastic/myeloproliferative neoplasia (MDS or MDS/MPN overlap), myeloproliferative neoplasm (MPN), and chronic myeloid leukemia (CML). Lymphoid malignancy included acute lymphocytic leukemia (ALL), chronic lymphocytic leukemia (CLL), Hodgkin lymphoma (HL), non-Hodgkin lymphoma (NHL) and plasma cell disorders (PCD) (which included multiple myeloma and others). Non-malignant disorders (NMD) included bone marrow failure (BMF: severe aplastic anemia (SAA) and other BMF), thalassemia and sickle cell disease (HG), primary immune deficiencies (PID), inherited diseases of metabolism (IDM), and autoimmune diseases (AID). Others included histiocytosis and other rare disorders not included in the above.

## Results

### Participating centers in 2020

Of the 690 centers, 459 (66%) performed both allogeneic and autologous transplants; 212 (31%) restricted their activity to autologous HCT, and 15 (2%) to allogeneic transplants only. Four of the 690 responding centers reported no activity due to renovation or changes within the transplant unit. Within the 686 actively transplanting centers in 2020, 123 (18%) performed transplants on both adult and pediatric patients. An additional 124 (18%) were dedicated pediatric transplant centers and 439 (64%) performed transplants on adults only. Twenty-nine centers failed to report in 2020, which, when compared with previously reported data, accounted for ~927 non-reported HCTs.

### Numbers of patients, transplants, and trends in 2020

**Fig. 1:**
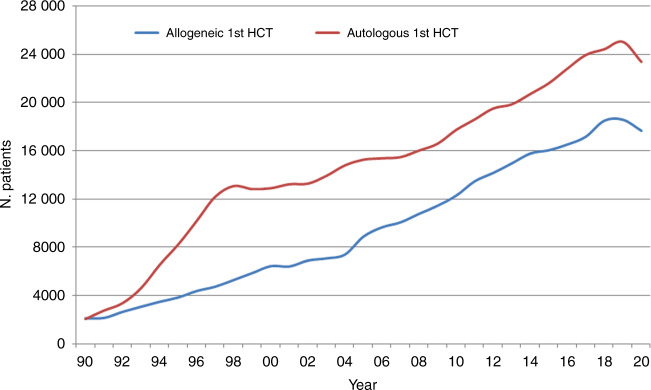
The absolute numbers of patients who received their first transplant during the years 1990–2020. Absolute number of patients receiving a 1st HCT 1990–2020.

In 2020, 45,364 transplants were reported in 41,016 patients (first transplant); of these, 18,796 HCT (41%) were allogeneic and 26,568 (59%) autologous (Table 1 and Fig. 1). Compared with 2019, the total number of transplants decreased by −6.5% (−5.1% allogeneic HCT and −7.5% autologous HCT) [14]. The corresponding number of patients showed a decrease of −4.9% for allogeneic HCT and −6.6% for autologous HCT. In addition, there were 4348 s or subsequent transplants, 1149 being allogeneic (−7%), mainly to treat relapse or graft failure and 3199 (−13%) autologous, the majority of which were likely to have been part of multiple transplant procedures such as tandem procedures, or as salvage autologous transplants for PCD. Furthermore, 753 of the allogeneic HCTs were reported as being given after a previous autologous HCT and were mainly for lymphoma or PCD.

### Pediatric transplantation

The number of pediatric patients (<18 years old at transplant) transplanted in both dedicated pediatric and joint adult-pediatric units was 5155 (3768 allogeneic and 1387 autologous). This is an overall decrease of −0.7% in the total number of transplants, with a decrease of −5.6% in allogeneic HCT but an increase of 15.7% in autologous HCT when compared to 2019. Of these, 3629 patients, (2746 allogeneic (75%) and 883 autologous (25%)) were treated in 124 dedicated pediatric centers in 27 countries. Due to the design of the survey, detailed analysis is limited to the dedicated pediatric activity only centers. Main indications for allogeneic HCT were AML (*n* = 388; 65% in early stage), ALL (*n* = 783; 46% in early stage) and NMD (*n* = 1236; 38% PID). There were 1529 family and 1217 unrelated donor HCTs reported. Within family donors, 45% were from a haploidentical relative. Bone marrow was used as the stem cell source in 1262 patients of which 65% were family donors. Peripheral blood stem cells were used in 1356 patients with similar proportions seen in both family (*n* = 684) and unrelated donors (*n* = 672). Cord blood stem cells were used in 128 pediatric patients of which 106 (83%) were from unrelated cord blood donors. The main indications for autologous HCT, were solid tumors, with 755 HCT reported in 2020, primarily for neuroblastoma (41%).

### Main indications

**Fig. 2:**
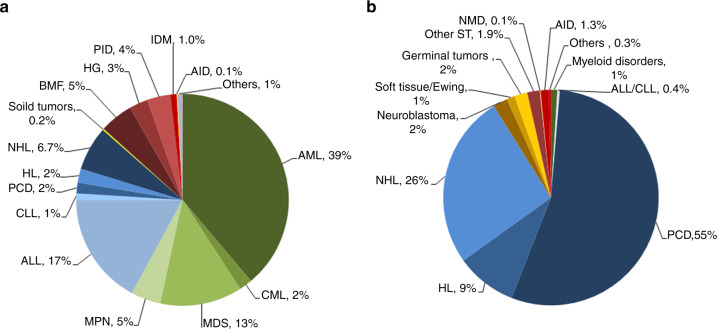
Relative proportion of disease indications for HCT in Europe 2020. **a** Relative proportion of allogeneic HCT. **b** Relative proportion of autologous HCT.

Indications for HCT in 2020 are listed in detail in Table 1 (Fig. 2a, b shows the distribution of disease indications for allogeneic (Fig. 2a) and autologous (Fig. 2b) HCT). Main indications for allogeneic HCT were myeloid malignancies (AML, CML, MDS or MDS/MPN overlap and MPN): 10,441 (98% allogeneic HCT and 2% autologous HCT). For autologous HCT, the main indications were lymphoid malignancies (ALL, CLL, PCD, HL and NHL): 26,120 (19% allogeneic HCT and 81% autologous HCT).

### Allogeneic HCT

**Fig. 3:**
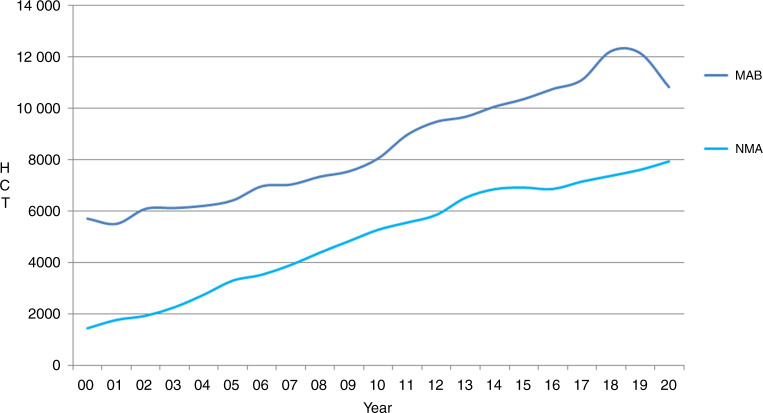
The change in the numbers of myeloablative versus non-myeloablative allogeneic HCT performed in Europe during the years 2000–2020. Change in the numbers of MAB versus NMA HCT in Europe 2000–2020.

This year, a decrease in activity was seen in the majority of disease indications. The leading indication for allogeneic HCT was AML, which accounts for 39% of all allogeneic HCT, a decrease of −2.1% when compared to 2019. Decreases were seen in all disease stages: first complete remission: −1.6%, therapy-related AML or those with myelodysplasia-related changes: −4.2% and in late disease stage: −2.1%. Among the myeloid malignancies, CML also decreased overall by −10.4%. Allogeneic HCT for the myeloproliferative syndromes decreased for both MDS by −4.3% and MPN by −1.2%. ALL comprised 17% of allogeneic HCT but only showed a slight decrease overall of −0.7% compared to the previous year. Again, differences were seen between early stage, decreasing by −0.9% and late stage, decreasing by −0.3%. Allogeneic HCT for CLL continued to decrease by −1.2% when compared to 2019, a constant trend seen over recent years although much less so between 2018 and 2020. A large decrease was seen for both allogeneic HCT for NHL of −9.2% and for HL of −13.6%. Within the NMD, a decrease of −9.7% was seen for BMF–SAA (*n* = 676), and of −17.2% for BMF–non SAA (*n* = 226). PID decreased by −13.6% (*n* = 621) and sickle cell disease by −30.9% (*n* = 224). For IDM, the rate remained stable with only a slight decrease of −1.1% (*n* = 173) and for thalassemia a decreased was seen of −19.6% (*n* = 275). Allogeneic HCT for AID remained a rare indication with just 18 patients treated in 2020. Within allogeneic HCT, 7956 were performed using non-myeloablative or reduced intensity conditioning in 2020. This comprised 42% of all allogeneic HCTs, of note, a remarkable decrease in transplants using myeloablative conditioning (−11%) but not non-myeloablative conditioning was observed (Fig. 3).

### Donor type and stem cell source

**Fig. 4:**
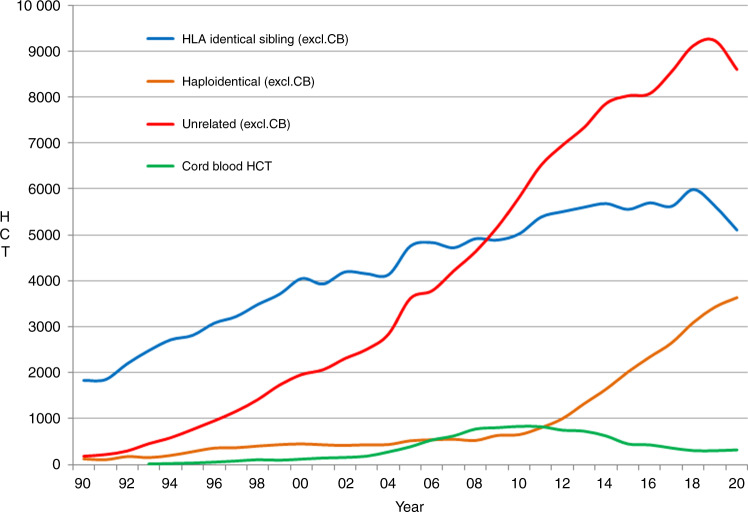
The changes seen in the choice of donor during the years 1990–2020. Change in choice of donor type from 1990 to 2020.

In 2020, as seen with transplant activity, changes were seen in the choice of donor. The overall numbers of patients treated with family donors decreased by −3.5%, however, variation was seen within the choice of family donor used. HLA identical sibling and syngeneic twin donors decreased by −9.3% when compared to 2019 but an increase was observed in haploidentical donors of 6.2%. For unrelated donors excluding unrelated cord blood HCT, a decrease of −6.8% was reported (Fig. 4). For the first time in several years, cord blood HCT rate for all donor types increased by 11.7% from 309 to 345 and mainly included unrelated cord blood (86%). In sibling donors, peripheral blood and bone marrow stem cell use decreased by −7% and −16% respectively. In haploidentical donors, an increase of 11.6% was seen in the use of stem cells harvested from peripheral blood while use of bone marrow stem cells decreased by −12.4%. In unrelated donor transplants, the use of bone marrow decreased by −37% and cord blood stem cells increased by 10%. There was a shift in allogeneic HCT from marrow toward peripheral blood as stem cell source. Use of marrow decreased by 16% for sibling donors, by 37% for unrelated donors and by 13% for haploidentical donors. This possibly reflected decreased availability of operating theaters during the SARS-Cov-2 pandemic and the technical difficulty to freeze and thaw allogenic marrow cells as compared to mobilized peripheral blood stem cells when cryopreservation was introduced as a means to mitigate the risks of donor unavailability in relation to SARS-CoV-2 infection.

### Autologous HCT

**Fig. 5:**
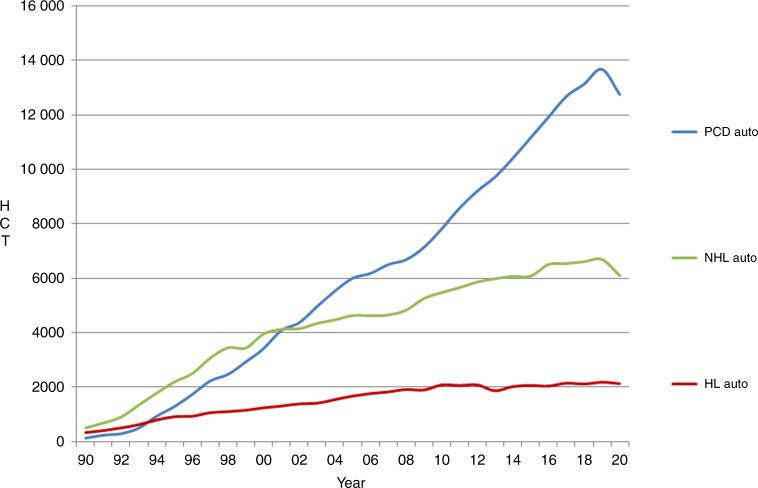
The change in the absolute numbers of autologous HCT reported for lymphoproliferative disorders during the years 1990–2020. Change in the absolute numbers of autologous HCT for lymphoproliferative disorders from 1990 to 2020.

As with allogeneic HCT, a decrease in activity was seen in the majority of disease indications. The main indications for autologous HCT were lymphoid malignancies (85%) with PCD comprising 55% of all autologous HCT patients. When compared to 2019, a decrease was seen in PCD (−6.8%), NHL (−8.9%), HL (−2.3%), ALL (−18.2%) and AML (−6.3%) (Fig. 5). For solid tumors, the numbers increased by 9% from 1547 to 1686. For AID, an overall decrease of −44.7% from 539 to 298 was seen. Twenty-five countries reported AID HCT in both 2019 and 2020, 17 of which reported a decrease in autologous HCT activity. The main decrease was seen in the numbers of HCT for multiple sclerosis (49% of all AID), from 442 to 226. This decrease, most likely related to the SARS-CoV-2 pandemic within the predominant countries of activity in this field, also reflected EBMT guidelines [28].

### Transplant rates

Assessing transplant rates per 10 million population (TR) allowed the comparison of activity in countries where population numbers differ greatly. In the 2020 survey, the TR rates for allogeneic HCT, ranged from 2.2 in Iraq, 5.3 for Latvia and Ukraine to 400 in Germany (median number of HCT 107 and TR 131.5). Four countries did not report any allogeneic HCT (Armenia, Cyprus, Luxembourg and Nigeria). For autologous HCT, rates ranged from 0.01 in Nigeria, 1.0 in Azerbaijan to 603 in Switzerland (median number of HCT 161 and TR 276).

### Cellular therapy

Table 2 shows the number of patients who received advanced cellular therapy and DLI performed in 2020. Un-manipulated DLIs were reported in 3055 patients, which is a slight decrease of −0.9% since 2019. The majority of DLIs were given for relapse (*n* = 1265) and graft enhancement/failure (*n* = 728).

**Fig. 6:**
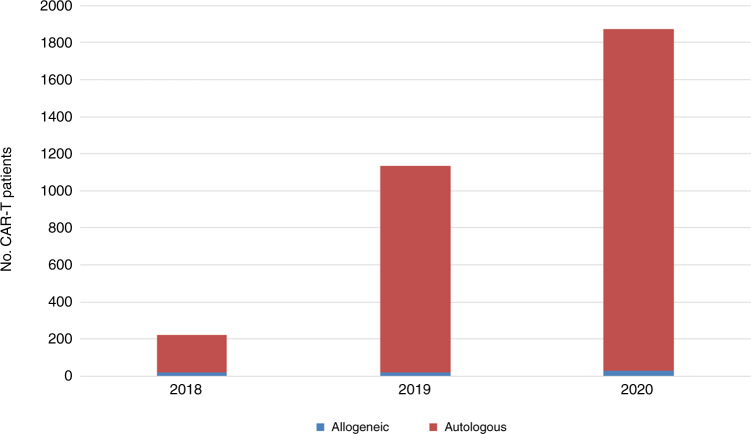
The absolute numbers of CAR-T therapies by donor type reported during the years 2018–2020. Absolute numbers of CAR-T therapies by donor type 2018–2020.

A total of 2920 patients in 251 centers from 29 countries received other forms of hematopoietic cellular therapies that qualified as medicinal products rather than cell transplants [16]. In 2020 the most remarkable increase seen was in gene-modified T cells, notably CAR-T cells, increasing from 151 in 2017 to 1875 in 2020. When compared to 2019, an increase of 65% from 1134 to 1875 CAR-T therapies was reported (Fig. 6).

One hundred and fifty-four centers in 22 countries reported CAR-T cellular therapies in 2020. The main indication was lymphoma (*n* = 1,437; 99% autologous), followed by ALL (*n* = 316; 92% autologous) and other malignancies (*n* = 122; 98% autologous). The second most widely used cellular therapy other than CAR-T cells in 2020 was mesenchymal stromal cells (*n* = 426; 97% allogeneic), their use being mainly to treat graft-versus-host disease [22]. Numbers of other cellular therapy products have not greatly changed since 2019. Specific data on tumor infiltrating lymphocytes is not collected in the annual survey.

### CAR-T rates

CAR-T rates per 10 million population in 2020 ranged from 1.1 in Belarus to 166 in Israel for both allogenic and autologous patients. The median number of CAR-T patients was 25 and TR 17.9. Eight countries (Belarus, France, Germany, Greece, Italy, Russia, Slovenia and Spain) reported a total of 29 patients with allogeneic CAR-T. For autologous CAR-T, 21 countries reported a total of 1846 CAR-T’s, the median being 24.5.

### Effect of the SARS-CoV-2 pandemic on activity

**Fig. 7:**
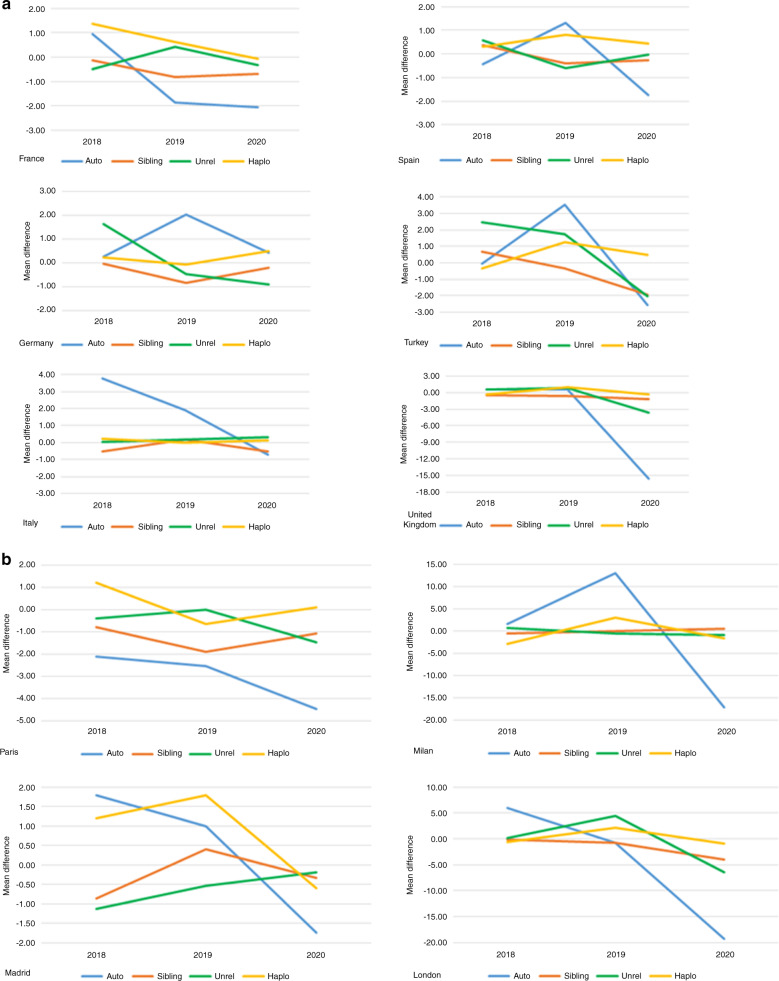
Average change in the number of transplants in centers reporting consistently over the 4-year period 2017–2020. Take note of the changing scale on the *Y*-axis. **a** Average change in the number of transplants in selected countries. **b** Average change in the number of transplants in selected cities with more than five reporting centers.

The first activity survey was conducted in 1990 and presented data on 4,234 patients reported by 143 centers in 21 countries. Since this first survey, revisions were made to ensure that each annual survey was optimized for the current changes in technology and treatment protocols used at the time. The survey allowed us to monitor activities and trends over the 31-year period. The number of reporting centers increased from 143 to 690 in 2020 and from 21 countries to 51. Transplant numbers increased from 4234 to 45,364. After 30 years of continuous growth, a decrease in annual activity was seen for the first time in both allogeneic and autologous HCT, as the medical community entered the first year of the SARS-CoV-2 pandemic. The decrease amounted to −6.5% of the overall transplant activity, whereas the use of CAR-T cell technology continued to increase by 65%. In autologous HCT a decrease was seen for all indications, while in allogeneic HCT the decrease was limited to unrelated donor and matched sibling donor transplantation but not the use of haploidentical donor transplantation, and similarly to the use of myeloablative conditioning but not non-myeloablative conditioning. To further elucidate we looked at the use of different transplant technologies in specific countries as shown in Fig. 7a. We could not observe a particular trending pattern other than the decrease in autologous HCT 2019–2020 being rather uniform. The graphic shows the average number of change from year to year per center in a selection of countries reporting consistently between 2017 and 2020. As cities were hit differently by the pandemic, we also show in Fig. 7b a few major cities with more than five reporting centers per city.

## Discussion

The EBMT activity survey has been conducted annually since 1990 [1]. Over 45,000 transplants in more than 41,000 patients were reported in 2020. The largest number of transplants ever reported was in 2019 (48,412 in 43,581 patients). Therefore, for the first time in 2020 a decrease in transplant activity was observed, most likely due to the SARS-CoV-2 pandemic, but also possibly due to the introduction of innovative therapies in hematology. Overall this decrease was moderate (−6.5%) given the extent health care systems were hit by the pandemic in the year 2020. Activity also reflected the regular updates and cautious guidance for clinical practice from EBMT “http://www.ebmt.org/covid-19-and-bmt” and national bodies. At the same time activity of cellular therapies, in particular CAR-T cells increased by 65% in 2020. It is important to stress that CAR-T cells represent a relatively recent therapeutic option for patients with lymphoid malignancies, while autologous and allogeneic HCT represent an established field of activity with indications refined over several decades. Restrictions associated with successive waves of SARS-CoV-2 infections—including lockdowns imposed at different times in countries in and outside of Europe—disturbed the supply chains for several categories of HCT and hematopoietic cellular therapies, and limited access to critical hospital facilities, such as ICU wards. The extent to which the pandemic endangered patient access to curative treatment cannot be precisely measured with this type of survey. Subsequent surveys may shed light on the question as to whether the changes shown here will prove to be temporary or permanent. Although we do not have data on the distribution of treatment activity during the year of 2020, some personal communication indicated that temporary slowing of the transplant programs early in 2020 was compensated later in the year. Of note was a decrease in autologous HCT for both NHL and myeloma possibly indicating that it is not CAR-T treatments replacing autologous HCT. This appears to be different to what was seen in the US where a shift from autologous HCT to CAR-T treatment has been reported [34, 35]. For allogeneic transplantation, a switch to less toxic conditioning regimens e.g., a drop in myeloablative but not in non-myeloablative regimens fits this picture, as is a slight drop in use of unrelated donors with a small increase in the use of haploidentical donors. Unrelated donor transplantation was difficult during the transportation restrictions caused by the pandemic; therefore the drop seems rather moderate. We cannot explain the decrease in the use of matched sibling donors as this was reported for all indications and was not restricted to resource poor countries. The shift from marrow toward use of peripheral blood for allogeneic HCT for all donor types, probably reflects institutional restrictions due to the SARS-CoV-2 pandemic and increasing use of cryopreservation of stem cell products rather than scientific concerns. For other changes, such as decreasing use of allogeneic HCT for CML, we cannot separate the effect of the SARS-CoV-2 pandemic from other trends such as the use of more potent kinase inhibitors. Likewise changes in the use of allogeneic HCT for NHL or HL may be related to availability of monoclonal and bispecific antibodies or checkpoint inhibitors. Increasing availability of targeting agents may also have changed transplant indications for AML. A more in depth analysis of some countries and major cities differentially hit by the SARS-CoV-2 pandemic did not provide a clear and uniform picture pointing to the fact that most transplant centers adapted their transplant program according to the situation. Obviously, we have reported on transplant activity and do not have data on patient outcome which may have been impacted by the SARS-CoV-2 pandemic and will be analyzed in other studies.

The annual activity survey of the EBMT reflects current activity and trends in the field of transplant technology. We showed a decrease in transplant activity ascribing the decrease in allogeneic HCT for non-malignant diseases and the decrease in autologous HCT for AID, the increased use of haploidentical donors and cord blood and the decrease in use of bone marrow and unrelated donors, the increase in non-myeloablative conditioning mostly to the SARS-CoV-2 pandemic. These were expected changes, reflecting the transplant practice in the midst of a pandemic with different national policies on lockdowns, personnel and logistic issues. Transplant physicians were probably choosing non-transplant approaches where acceptable. Use of DLIs used for relapses and poor-graft function remained stable. The increase in CAR-T cell activity reflects the wide adoption of a newly approved modality supported by academic activities. This report is valuable for the dissemination of the most recent information on indications, donor and stem cell usage and benchmarking [16], which will ultimately be beneficial in health care planning.

## Supplementary information

Supplementary data
