# Supplementary material for: Impact of the SARS-CoV-2 pandemic on hematopoietic cell transplantation and cellular therapies in Europe 2020: a report from the EBMT activity survey
Source: Bone Marrow Transplant. 2022 Feb 22;57(5):742–52. doi: 10.1038/s41409-022-01604-x (PMC8862400; doi:10.1038/s41409-022-01604-x)
Supplement: Supplementary file 1 — Supplementary data [file 41409_2022_1604_MOESM1_ESM.pdf]

EBMT

APPENDIX 2020

EBMT Transplant Activity Survey: List of reporting transplant centers in 2020

(Number of reporting centers: total 1st HCT (total all HCT) N allogeneic 1st HCT / N autologous 1st HCT)

Algeria: (2 teams: 213 (218) 110/103)
Alger, Centre Pierre et Marie Curie, (ads, peds), CIC 703, R. Hamladji (147 (152) 100/47)
Oran, University Hospital of Oran, (ads, peds), CIC 993, M. Bekadja (66 (66) 10/56)

Armenia: (1 team: 15 (15) 0/15)
Yerevan, Hematology Centre after Prof. R. Yeolyan, (ads, peds), K. Meliksetyan, S. Dagbashyan (15 (15) 0/15)

Austria: (13 teams: 565 (618) 236/329)
Graz, University of Graz, (ads), CIC 308, H. Greinix, H. Sill (92 (96) 44/48)
Graz, Universitäts Kinderklinik, (peds), CIC 593, C. Urban, W. Schwinger (6 (6) 4/2)
Innsbruck, University Hospital, (ads, peds), CIC 271, D. Wolf, D. Nachbaur (76 (81) 39/37)
Klagenfurt, Klinikum Klagenfurt, (ads), S. Eder (43 (43) 9/34)
Linz, Kepler University Hospital, (ads), CIC 343, C. Schimitt (4 (4) 0/4)
Linz, AOKH der Elisabethinen, (ads), CIC 594, J. Clausen, M. Binder (110 (130) 56/54)
Salzburg, LKA Salzburg, (ads), CIC 356, R. Greil, G. Rass (27 (29) 0/27)
St. Pölten, University Hospital St. Pölten, (ads), CIC 474, G. Krajnik, Ch. Fertl (18 (19) 0/18)
Vienna, St. Anna Kinderspital, (peds), CIC 528, H. Pichler,W. Holter, S. Karlhuber (24 (32) 18/6)
Vienna, Donauspital, (ads), CIC 767, C. Sebesta, P. Kier (9 (12) 0/9)
Vienna, Universitätsklinik fur Innere Medizin-AKH, (ads), CIC 227, W. Rabitsch (100 (105) 66/34)
Vienna, Kilinik Ottakring, Wilhelminenspital, (ads), CIC 828, W. Hilbe, N. Zojer (29 (33) 0/29)
Vienna, Hanusch- Krankenhaus, (ads), CIC 743, F. Keil (27 (28) 0/27)

Azerbaijan: (1 team: 16 (16) 15/1)
Baku, Republican Thalassemia Centre, (peds), CIC 176, N. Aliyeva Rafiq, S. Saila Rauf (16 (16) 15/1)

Republic of Belarus: (2 teams: 163 (169) 40/123)
Minsk, Belorussian Center, (peds), CIC 591, O. Aleinikova, Y. Mareika (29 (35) 18/11)
Minsk, Scientific Centre of Surgery, Transplantology and Hematolgy, (ads), N. Milanovich (134 (134) 22/112)

Belgium: (17 teams: 838 (924) 395/443)
Antwerp, Stuivenberg ZH, (ads), CIC 339, W. Ka Lung (23 (23) 12/11)
Brussels, University Hospital, (ads), CIC 630, R. Schots, F. Trullemans (48 (51) 18/30)
Brussels, U.L.B. Hôpital Erasme, (ads), CIC 596, V. De Wilde (13 (15) 0/13)
Brussels, Clinique Universitaire St. Luc, (ads, peds), CIC 234, X. Poiré, C. Vermylen (67 (68) 52/15)
Brugge, A.Z. St. Jan, (ads), CIC 506, D.Selleslag, J.v.Droogenbroeck (76 (78) 37/39)
Brussels, Institut Jules Bordet and the Childrens Hospital, (ads, peds), CIC 215, P. Lewalle, D. Bron, C. Devalck (81 (84) 51/30)
Charleroi, Grand Hôpital de Charleroi Notre-Dame, (ads), CIC 349, D. Pranger (11 (12) 0/11)
Antwerp, University Antwerpen, (ads), CIC 996, W. Schroyens, Z. Berneman (68 (74) 37/31)
Gent, University Hospital, (ads, peds), CIC 744, C. Dhooge, T. Kerre, V. Bordon (94 (103) 43/51)
Haine St. Paul, Hôpital de Jolimont, (ads), CIC 2343, H. Petre, F. Van Obbergh (21 (26) 0/21)
Hasselt, Jessa Ziekenhuis, (ads), CIC 632, K. Theunissen (31 (38) 0/31)
Leuven, University Hospital Gasthuisberg and Leuven, (ads, peds), CIC 209, J. Maertens,G.Verhoef, A. Uyttebroeck, M. Renard (122 (136) 64/58)
Liège, University Hospital Sart-Tilman, (ads, peds), CIC 726, Y. Béguin, B de Prijck (70 (84) 38/32)
Roeselare, AZ Delta, H. Hart Ziekenhuis, (ads), CIC 646, D. Deeren (39 (46) 15/24)
Turnhout, AZ Turnhout, (ads), CIC 910, I. Vrelust, B. Hodossy (6 (6) 0/6)
Wilrijk-Antwerp, St. Agustinus Hospital, (ads), CIC 715, J. Lemmens, C. Schuermans (10 (13) 0/10)
Yvoir, Clinique universitaire de Mont-Godinne, (ads), CIC 2342, C. Doyen (58 (67) 28/30)

Bosnia-Herzegovina: (2 teams: 36 (41) 3/33)

Sarajevo, Clinical Centre, (ads), CIC 198, A. Sofo-Hafizovic (29 (34) 0/29)
Tuzla, University Clinical Centre, (ads), H. Sahovic (7 (7) 3/4)

Bulgaria: (2 teams: 123 (126) 51/72)
Sofia, University Hospital Queen Johanna-Isul, (peds), CIC 346, D. Konstantinov, B. Avramova (10 (11) 1/9)
Sofia, National Centre of Hematology, (ads), CIC 859, B. Spassov, G. Arnaudov, K. Simeonova (113 (115) 50/63)

Croatia: (3 teams: 240 (287) 93/147)
Zagreb, Hospital Merkur, (ads), CIC 466, D. Radic-Kristo, V. Zatezalo (43 (65) 0/43)
Zagreb, University Hospital Rebro, (ads, peds), CIC 302, R. Vrhovac, R. Serventi-Seiwerth (182 (202) 93/89)
Zagreb, University Hospital Dubrava, (ads), CIC 407, V. Pejsa, O. Jaksic, Z. Prka (15 (20) 0/15)

Cyprus: (1 team: 31 (31) 0/31)
Nicosia, Nicosia General Hospital, (ads), CIC 575, K. Melanthiou, C. Stylianou (31 (31) 0/31)

Czech Republic: (9 teams: 554 (686) 215/339)
Brno, Masaryk University Hospital, (ads, peds), CIC 597, J. Mayer, Z. Racil, J. Sterba (77 (102) 25/52)
Hradec Kralové, Charles University Hospital, (ads), CIC 729, P. Zak (71 (78) 34/37)
Olomouc, University Hospital, (ads), CIC 574, E. Faber (48 (57) 18/30)
Ostrava, Universtity Hospital Ostrava, (ads), CIC 156, R. Hajek (41 (62) 1/40)
Pilsen, Charles Hospital, (ads), CIC 718, P. Jindra, A. Jungova (103 (129) 34/69)
Prague, Charles University Hospital, (ads), CIC 318, T. Kozak (33 (38) 0/33)
Prague, University Hospital Motol, (peds), CIC 452, P. Sedlacek (45 (56) 30/15)
Prague, Charles University, (ads), CIC 745, M. Trneny (63 (82) 0/63)
Prague, Institute of Hematology and Blood Transfusion, (ads), CIC 656, A. Vitek (73 (82) 73/0)

Denmark: (3 teams: 325 (354) 163/162)
Aalborg, Aalborg Hospital, (ads), CIC 848, I. Christiansen, J. Baech, K.Nielsen (no report)
Aarhus, Aarhus Amtssygehus, (ads, peds), CIC 634, M. Thorsgaard (117 (130) 53/64)
Copenhagen, Rigshospitalet, (ads, peds), CIC 206, H. Sengelov, C. Heilmann (184 (190) 110/74)
Copenhagen, Herlev Hospital, (ads), CIC 568, P. Josefsson, P. Andersen (24 (34) 0/24)

Estonia: (2 teams: 85 (96) 26/59)
Tallinn, North Estonia Medical Centre, (ads), CIC 984, K. Palk (40 (46) 0/40)
Tartu, University Hospital, (ads, peds), CIC 746, A.Kaare (45 (50) 26/19)

Finland: (7 teams: 381 (407) 139/242)
Helsinki, Helsinki University Hospital, (ads), CIC 833, S. Mannisto (18 (18) 0/18)
Helsinki, Children's Hospital, (peds), CIC 219, K. Vettenranta (25 (29) 23/2)
Helsinki, Helsinki University Central Hospital, (ads, peds), CIC 515, R. Niittyvuopio (98 (107) 64/34)
Kuopio, University Hospital, (ads, peds), CIC 396, A. Partanen (54 (60) 0/54)
Oulu, Oulu University Central Hospital, (ads), CIC 690, M. Säily (49 (52) 0/49)
Tampere, University Hospital, (ads, peds), CIC 635, M. Sankelo, M. Sinisalo (45 (49) 0/45)
Turku, University Central Hospital, (ads, peds), CIC 225, U. Salmenniemi, M. Itäla-Remes (92 (92) 52/40)

France: (71 teams: 4,327 (4,753) 1,770/2,557)
Amiens, CHU d'Amiens, (ads), CIC 955, A. Charbonnier, J.P. Marolleau (86 (86) 43/43)
Angers, Centre Hospitalier, (ads, peds), CIC 650, M. Hunault-Berger, S. Francois (72 (90) 29/43)
Arras, Groupe Hospitalier Artois Ternois, (ads), CIC 999, P. Lionne-Huyghe (9 (10) 0/9)
Argenteuil, Hopital Victor Dupouy, (ads), CIC 199, A. Al Jijakli (13 (19) 0/13)
Bayonne, C.H. De la Cote Basque, (ads), S. Labarrere, A. Banos (34 (35) 0/34)
Besancon, Hopital Jean Minjoz and St.Jacques, (ads, peds), CIC 233, E.Deconinck (76 (77) 36/40)
Bordeaux, CHU Hopitalier Pellegrin- Enfants, (peds), CIC 978, C. Jubert, Y. Perel (31 (36) 24/7)
Brest, Hopital Morvan, CHU de Brest, (ads, peds), S. Creachcadec, G. Guillerm, C. Berthou (77 (93) 23/54)

Caen, CHU Caen Institut d’hématologie de Basse-Normandie CHU, (ads, peds), CIC 251, G. Damaj,S. Chantepie (78 (83) 29/49)
Colmar, Louis Pasteur Hospital, ads, JM. Limacher (no report)
Le Chesnay, Hôpital André Mignot, Varsailles, (ads), Ph. Rousselot, S. Castaigne (42 (42) 0/42)
Clermont Ferrand, CRCTCP, CHU Estaing, (ads, peds), CIC 273, J.-O. Bay (113 (128) 49/64)
St. Cloud, Institut Curie, Hopital René Huguenin, (ads), S. Glaisner (32 (36) 0/32)
Corbeil Essonne, Hopital Gilles de Corbeil, (ads), A. Devidas, S. Haiat (18 (21) 0/18)
Créteil, Hopital Henri Mondor, (ads), CIC 252, C. Cordonnier, S. Maury (44 (50) 44/0)
Créteil, Hopital Henri Mondor, (ads), CIC 432, C. Haioun, J. Dupuis (38 (39) 0/38)
Dijon, Hopital des Enfants, (ads), D. Caillot (76 (93) 0/76)
Dunkerque, Centre Hospitalier, (ads), J-M. Pignon, M. Wetterwald (15 (15) 0/15)
Saint Priest en Jarez, Institut de Cancérologie Lucien Neuwirth, (ads), CIC 250, D. Guyotat, J. Cornillon (55 (59) 27/28)
Grenoble, CHV Grenoble Alpes, (ads, peds), CIC 270, J.Y. Cahn, C.E. Bulabois (104 (119) 49/55)
Lens, Service d'Hématologie, (ads), C. Bories, L. Stalnikiewicz (19 (19) 0/19)
Limoges, CHU Dupuytren, (ads), CIC 977, P. Turlure, D. Bordessoule (44 (46) 17/27)
Lille, Centre Hospitalier Saint Vincent, (ads), B. Carpentier, L. Pascal (20 (20) 0/20)
Lille, Centre Oscar Lambret, (peds), A. Defachelles (11 (17) 0/11)
Lille, Hopital Claude Huriez, (ads), CIC 277, I. Yakoub-Agha (101 (111) 98/3)
Lille, Hopital Jeanne de Flandre, (peds), CIC 963, B. Bruno, B. Nelken (19 (21) 19/0)
Lyon, Centre Hospitaier Lyon Sud, (ads), CIC 671, H. Labussiere-Wallet (80 (83) 79/1)
Lyon, Centre Léon Bérand, (ads), CIC 241, E. Nicolas-Virelizier (58 (70) 0/58)
Lyon, Institue d'Hématologie et d'Oncologie Pédiatrique, (peds), CIC 806, Y. Bertrand (30 (35) 19/11)
Marseille, Institut Paoli I. Calmettes, (ads), CIC 230, D. Blaise, C. Chabannon (206 (233) 105/101)
Marseille Bouches du Rhone, Centre Hospitalier Universitaire La Conception, (ads), CIC 158, R. Costello (23 (23) 0/23)
Marseille, Hopital Timone Enfants, (peds), CIC 301, G. Michel, C. Coze (38 (45) 24/14)
Meaux, CHU de Meaux, (ads), CIC 194, J. Frayfer, L. Fouillard (13 (16) 0/13)
Montpellier, CHR Lapeyronie and CHU Arnaud de Villeneuve, (ads, peds), CIC 926, N. Fegueux, A. Sirvent (194 (198) 62/132)
Mulhouse, Hopital E. Muller, (ads), CIC 944, B. Drénou, M. Ojeda-Uribe (27 (33) 0/27)
Nantes, CHU Nantes, (ads, peds), CIC 253, P. Chevallier, S. Legouill, F. Rialland (173 (187) 84/89)
Nice, Hopital de l'Archet, (ads, peds), CIC 523, P. Rohrlich, T. Cluzeau (54 (55) 42/12)
Nice, Centre Antoine Lacassagne, (ads), CIC 973, A. Thyss (64 (71) 0/64)
Orleans, CHR Orléans, (ads), M. Alexis, C. Benbrahim, O. Michel (19 (21) 0/19)
Paris, Immuno-Hèmatologie, Hôpital St. Louis, (ads), B. Arnulf (64 (75) 0/64)*
Paris, Hôpital Neckar des enfants malades, (peds), CIC 201, B. Neven, S. Blanche (38 (39) 38/0)
Paris, Hôpital St. Louis, (ads), CIC 805, C. Thieblemont (24 (24) 0/24)
Paris, Hôpital St. Louis, (ads, peds), CIC 207, G. Socié, M. Robin, R Peffault de La Tour (127 (128) 119/8)
Paris, Hôpital St. Antoine, (ads), CIC 775, M. Mohty, S. Lapusan (92 (108) 50/42)
Paris, Hôpital Pitié Salpétière, (ads), CIC 262, V. Leblond, S. Nguyenquoc (79 (83) 38/41)
Paris, Hôpital Tenon, (ads), CIC 747, J.P. Lotz, F. Selle (11 (23) 0/11)
Paris, Hopital d'enfants Armand Trousseau, (peds), G. Leverger, A. Auvrignon (5 (5) 0/5)
Paris, Clarmart, Hopital d'Instruction des Armées Percy, (ads), JV. Malfuson (51 (53) 23/28)
Paris, Hôpital Robert Debré, (peds), CIC 631, JH. Dalle, A. Baruchel (60 (61) 60/0)
Paris, Hôpital Necker, (ads), CIC 160, O. Hermine, F. Suarez (75 (75) 48/27)
Paris, Institut Curie, (peds), CIC 702, Ph. Brault (21 (35) 0/21)
Paris, Hôpital Cochin, (ads), D. Bouscary (no report)
Pessac, Hôpital du Haut Leveque, CHU Bordeaux, (ads), CIC 267, N. Milpied,E. Forcade (190 (195) 79/111)
Lyon, Hospices Civils de Lyon, (ads), CIC 901, G. Salles (79 (87) 0/79)
Poitiers, CHU de Poitiers, Hôpital La Miletrie, (ads, peds), CIC 264, X. Leleu, M. Maillard, (126 (134) 43/83)
Pontoise, Hospital René Dubos, (ads), H. Gonzalez, I. Vaida (no report)
Rennes, CHU Rennes, (ads), CIC 6611, T. Lamy, M Bernard (105 (110) 39/66)
Reims, Hop. Robert Debré, (ads), CIC 959, A. Delmer, C. Himberlin (31 (33) 0/31)
Rennes, Clinique Médical Infantil, CHRU, (peds), CIC 6612, V. Gandemer (13 (13) 8/5)
Roubaix, Hopital V. Provo, (ads), I. Plantier-Colcher (13 (13) 0/13)
Rouen, Centre Henri Becquerel, (ads), CIC 941, H. Tilly, N. Contentin (76 (77) 27/49)

Rouen, Hopital Charles Nicolle, (peds), P. Schneider, N. Buchbinder (16 (22) 10/6)
Saint Quentin, Centre Hospitalier De Saint Quentin, (ads), CIC 406, R. Garidi (8 (9) 0/8)
La Réunion, CHU Felix Guyon, Saint Denis Centre, (ads), S. Vanderbecken (12 (12) 0/12)
Strasbourg, Nouvel Hopital Civil, (ads, peds), CIC 672, B. Lioure, .E. Kurtz, P. Lutz (124 (126) 55/69)
La Réunion, St. Pierre CHU Sud La Réunion, (ads), C. Mohr, C. Garnier (12 (15) 0/12)
Toulouse, Institut Universitaire du Cancer Toulouse Oncopole, (ads), CIC 624, A. Huynh, C. Recher (209 (220) 75/134)
Tours, Hôpital Bretonneau, (ads, peds), CIC 272, E. Gyan, L. Sutton (84 (84) 17/67)
Toulouse, Hopital Purpan, (peds), H. Rubie, G. Alphonsa (8 (13) 0/8)
Troyes, Centre Hospitalier de Troyes, (ads), CIC 472, A. Santagostino (19 (19) 0/19)
Vandoeuvre-les-Nancy, Hôpital d'Enfants and Brabois, (ads, peds), M. Rubio, P. Feugier, A. Campidelli, C. Pochon (127 (134) 73/54)
Valenciennes, Centre Hosp. de Valenciennes, (ads), M. Simon, N. Cambier (14 (16) 0/14)
Villejuif, Gustave Roussy Cancer Campus, (ads), CIC 666, J-H. Bourhis, C. Castilla-Llorente (164 (210) 66/98)
Villejuif, Institut Gustave Roussy, (peds), CIC 503, D. Valteau-Couanet (44 (67) 0/44)

Georgia: (1 team: 41 (41) 3/38)
Tbilisi, High Technology Medical Center, (ads), CIC 471, G. Ingorokva (41 (41) 3/38)

Germany: (114 teams: 6,774 (7,836) 3,079/3,695)
Aachen, Universitätsklinikum, (ads), T. Brümmendorf, E. Jost (90 (98) 49/41)
Aachen, Universitätsklinikum, (peds), CIC 348, U. Kontny (9 (10) 5/4)
Augsburg, Klinikum Augsburg, (ads, peds), CIC 152, C. Schmid (67 (73) 37/30)
Bad Saarow, Humaine Klinikum, (ads), R. Ratei (18 (18) 0/18)
Bamberg, Klinikum am Bruderwald, (ads), R. Seggewiss-Bernhardt (16 (26) 0/16)
Berlin, HELIOS Klinikum Berlin Buch, (ads), CIC 518, H. Baurmann (96 (109) 53/43)
Berlin, Charité, Campus Virchow Klinikum, (peds), CIC 336, J. Schulte (38 (42) 32/6)
Berlin, Vivantes Klinikum Neukoelln, (ads), CIC 105, M de Wit. L. Marretta (32 (36) 0/32)
Berlin, Charite, Campus Virchow Klinikum, (ads), CIC 807, I. Blau (255 (304) 115/140)
Bielefeld, Klinikum Bielefeld, (ads), CIC 949, M. Görner (16 (16) 0/16)
Bielefeld, Evangelisches Klinikum Bethel, (ads), CIC 116, F. Weissinger (1 (1) 0/1)
Bonn, Universitätsklinikum, (peds), CIC 403, D. Dilloo (5 (5) 4/1)
Bochum, Knappschaftskrankenhaus, (ads), CIC 124, R. Schroers (121 (151) 47/74)
Bonn, Universitätsklinikum, (ads), CIC 134, P. Brossart (72 (79) 37/35)
Bonn, Johanniter-Krankenhaus und Waldkrankenhaus, (ads), C. Sippel (38 (44) 0/38)
Bremen, Klinikum Bremen-Mitte, (ads), CIC 602, B.Hertenstein (38 (45) 11/27)
Bremen, Evangelistisch Diakonie-Krankenhaus GmbH, (ads), CIC 111, R.U.Trappe, N. Winkelmann (17 (25) 0/17)
Braunschweig, Städtisches Klinikum, (ads), CIC 674, J. Krauter (50 (55) 0/50)
Chemnitz, Klinikum Chemnitz GmbH, (ads), CIC 104, M. Hänel, A.Morgner (43 (51) 5/38)
Cottbus, Carl-Thiem-Klinikum, (ads), CIC 102, M. Schmidt-Hieber (15 (18) 0/15)
Dortmund, St. Johannes Hospital, (ads), CIC 125, R. Meyer, M.Hindahl (32 (37) 12/20)
Dresden, Universitätsklinikum Carl Gustav Carus, (ads), CIC 808, M. Bornhäuser, J. Schetelig (174 (203) 108/66)
Dresden, Universitätsklinikum Carl Gustav Carus, (peds), CIC 808, J. Hauer (9 (12) 7/2)
Duisburg, HELIOS Klinikum, (ads), CIC 519, C. Aul (53 (63) 20/33)
Düsseldorf, Universitätsklinikum, (peds), CIC 651, A. Borkhardt, R.Meisel (24 (29) 19/5)
Düsseldorf, Heinrich Heine Universitätsklinikum, (ads), CIC 390, G. Kobbe (106 (129) 53/53)
Erlangen, Universitäts Klinik für Kinder und Jugendliche, (peds), CIC 809, K. D. Stachel (8 (8) 6/2)
Erlangen, Universitätsklinikum, (ads), CIC 809, A. Mackensen, W. Rösler, J. Winkler (86 (95) 44/42)
Erfurt, Helios-Klinikum, (ads), CIC 966, H. Sayer, V. Schmidt (32 (36) 0/32)
Essen, West German Cancer Center, (ads), M. Schuler, S. Bauer (8 (13) 0/8)
Essen, Evangelisches Krankenhaus Essen-Werden GmbH, (ads), CIC 784, P.Reimer, M. Wattad (51 (54) 16/35)
Essen, Universitätsklinikum, (peds), CIC 259, R. Beier, O. Basu (20 (22) 13/7)
Essen, Universitätsklinikum, (ads), CIC 259, D.W. Beelen, H. Ottinger (223 (247) 149/74)
Flensburg, St. Franziskus Hospital, (ads), CIC 970, N. Basara, H. Menzel (37 (38) 21/16)
Freiburg, Universitatsklinikum, (peds), CIC 810, C. Niemeyer, B. Strahm (32 (33) 28/4)
Freiburg, Universitätsklinikum, (ads), CIC 810, J.Duyster, J. Finke, M.Engelhardt (214 (236) 77/137)

Frankfurt, Krankenhaus Bethanien, (ads), CIC 193, W. Knauf (17 (21) 0/17)
Frankfurt, Klinikum Frankfurt Oder, (ads), CIC 190, M. Kiehl (31 (36) 19/12)
Frankfurt, J. W. Goethe Universität, (peds), CIC 138, T. Klingebiel, P.Bader (37 (41) 30/7)
Frankfurt, Universitätsklinikum d. J. W. Goethe, (ads), CIC 297, H. Martin, H. Serve (117 (139) 69/48)
Giessen, Universitätsklinikum, (ads), CIC 463, A. Burchardt, M. Rummel (26 (29) 0/26)
Giessen, Universitätsklinikum, (peds), CIC 326, C. Mauz-Körholz (19 (20) 11/8)
Göttingen, Universitätsklinikum, (ads), CIC 552, G. Wulf, L. Trümper (141 (164) 75/66)
Greifswald, Universitätsklinikum, (ads), CIC 530, W. Krüger (41 (47) 18/23)
Greifswald, Universitätsklinikum, (peds), CIC 908, H. Lode (6 (7) 4/2)
Hamm, Evangelisches Krankenhaus, (ads), CIC 509, E.Lange (22 (28) 1/21)
Hannover, Klinikum Siloah, (ads), CIC 342, K. Marienhagen (36 (46) 0/36)
Halle, Universitätsklinikum, (peds), CIC 654, J. Klusmann, K.Kafa (7 (7) 4/3)
Hamm, St. Barbara-Klinik, (ads), CIC 470, H. Duerk (24 (30) 0/24)
Hagen, St. Marien Hospital, (ads), CIC 536, H. W. Lindemann (16 (26) 0/16)
Hannover, Medizinische Hochschule, (ads), CIC 295, A. Ganser (122 (133) 94/28)
Hamburg, Asklepios Klinik Altona, (ads), CIC 366, H. Salwender (43 (57) 0/43)
Hamburg, Universitätsklinikum Eppendorf - Onkologisches Zentrum, (ads), CIC 673, C. Bokemeyer (70 (78) 0/70)
Hamburg, Asklepios Klinik St. Georg, (ads), CIC 153, A. Elmaagacli (79 (84) 45/34)
Hannover, Medizinische Hochschule, (peds), CIC 295, C. Kratz, KW. Sykora (32 (32) 23/9)
Halle, Universitätsklinikum, (ads), CIC 338, L. Müller,T.Weber (82 (92) 44/38)
Hamburg, Universitätsklinikum Eppendorf, (ads), CIC 614, N.Kröger (186 (216) 166/20)
Hamburg, Unversitätsklinikum Eppendorf, (peds), CIC 882, I. Müller (51 (61) 42/9)
Heidelberg, Universitätsklinikum, (ads), CIC 524, C. Müller-Tidow, P. Dreger (244 (309) 88/156)
Heidelberg, Angelika Lautenschläger-Klinik, (peds), CIC 524, A. Kulozik, J. Greil (11 (11) 8/3)
Homburg/Saar, Universität des Saarlandes, (peds), CIC 7852, N. Graf, T. Krenn (3 (3) 0/3)
Homburg/Saar, Universität des Saarlandes, (ads), CIC 7851, N. Bittenbring (90 (106) 40/50)
Jena, Universitätklinikum, (ads), CIC 533, I. Higendorf, A. Hochhaus (93 (104) 43/50)
Jena, FS Universitäts-Kinderklinik, (peds), CIC 750, J. Beck, B. Gruhn (10 (16) 7/3)
Karlsruhe, Städtische Klinik, (ads), CIC 290, M.Bentz, M. Ringhoffer (58 (69) 25/33)
Kaiserslautern, Westpfalzklinikum, (ads), CIC 357, G. Held (15 (18) 0/15)
Kassel, Klinikum Kassel, (ads), M. Wolf, E. Steinhauer, M. Nathrath (18 (28) 0/18)
Kiel, Städtisches Krankenhaus, (ads), CIC 895, R. Repp (30 (31) 0/30)
Kiel, Universitätsklinikum Schleswig-Holstein, (ads), CIC 256, M.Gramatzki, T. Valerius (68 (81) 36/32)
Kiel, Universitätsklinikum Schleswig-Holstein, (peds), CIC 256, M. Schrappe (no report)
Köln, Universitätsklinikum, (ads, peds), CIC 534, M. Hallek,Ch. Scheid (184 (208) 91/93)
Koblenz, Stiftungsklinikum Mittelrhein, (ads), CIC 879, D.Niemann (20 (23) 0/20)
Leipzig, Universitätsklinikum, (ads), CIC 389, U. Platzbecker (149 (164) 71/78)
Leipzig, Universitätsklinikum, (peds), CIC 389, H. Christiansen (13 (13) 13/0)
Lemgo, Klinikum Lippe, (ads), F. Hartmann, C.Constantin (18 (24) 0/18)
Lübeck, Universitätsklinikum Schleswig Holstein, (peds), CIC 3672, M. Lauten (1 (1) 0/1)
Lübeck, Universitätsklinikum Schleswig Holstein, (ads), CIC 3671, F. Wortmann (46 (46) 23/23)
Ludwigshafen, klinikum der Stadt, (ads), CIC 140, M.Hoffmann (12 (15) 0/12)
Magdeburg, Universitätsklinikum, (ads), CIC 359, T. Heinicker,R. Bartsch (41 (46) 16/25)
Mannheim, Universitätsklinikum, (ads), CIC 142, W K. Hofmann, S. Klein (42 (51) 20/22)
Mainz, Universitätsklinikum, (ads), CIC 786, E. Wagner-Drouet, M. Theobald (165 (173) 103/62)
Marburg, Philipps Universitätsklinikum, (ads), CIC 645, A. Neubauer, A. Burchert (115 (128) 63/52)
Meschede, Klinikum Hochsauerland GmbH, (ads), M. Wattad, E. Lange (4 (5) 0/4)
Minden/Westfalen, Klinikum Minden, (ads), CIC 113, H. Tischler (48 (55) 0/48)
Möchengladbach, Klinikum Maria Hilf ll, KH St. Franziska, (ads), CIC 120, U.Graeven (6 (9) 0/6)
Münster, Universitätklinikum, (ads), CIC 680, M. Stelljes (252 (298) 141/111)
Munich, Klinikum Schwabing, (ads), CIC 151, A. Hausmann (56 (60) 25/31)
Munich, Haunersches Kinderspital Klinikum Grosshadern, (peds), CIC 513, M. Albert (34 (40) 31/3)
Munich, Rotkreuz Klinikum, (ads), CIC 883, M. Hentrich (70 (97) 0/70)
Munich, Universitätsklinikum Grosshadern, (ads), CIC 513, J.Tischer (95 (98) 52/43)
Münster, Universitätklinikum Münster, (peds), CIC 505, C. Rössig (34 (39) 25/9)
Munich, Klinikum Rechts der Isar, (ads), CIC 558, M. Verbeek (100 (124) 46/54)
Munich, Klinikum Schwabing, (peds), CIC 189, S.Burdach, A.Wawer (12 (12) 11/1)
Nürnberg, Klinikum Nuernberg, (ads), CIC 625, M.Wilhelm, K. Schäfer-Eckart (71 (79) 38/33)
Oldenburg, Universitätsklinikum, (ads), CIC 749, B. Metzner, CH.Köhne, J. Caspar (81 (86) 27/54)

Osnabrück, Klinikum Osnabrück, (ads), CIC 101, R.Peceny (29 (35) 0/29)
Potsdam, Klinikum Ernst von Bergmann, (ads), CIC 106, G. Maschmeyer (34 (44) 0/34)
Regensburg, Universitätsklinikum, (ads), CIC 787, E. Holler (152 (167) 69/83)
Regensburg, Universitätsklinikum, (peds), CIC 787, S. Corbacioglu (16 (17) 15/1)
Rostock, Universitätsklinikum, (ads), CIC 585, C. Junghanss (44 (65) 12/32)
Wümme, Diakoniekrankenhaus Rotenburg, (ads), CIC 871, F.Heits (22 (22) 0/22)
Schwerin, Helios Klinik Schwerin, (ads), CIC 447, A. Günther (25 (35) 11/14)
Siegen, St. Marien-Krankenhaus, (ads), CIC 135, R. Naumann (24 (26) 0/24)
Stuttgart, Diakonissen Krankenhaus, (ads), CIC 146, J. Greiner, S. Von Harsdorf (49 (67) 21/28)
Stuttgart, Robert Bosch Krankenhaus, (ads), CIC 145, S. Martin, W. Aulitzky, M.Kaufmann (72 (72) 26/46)
Stuttgart, Universitätsklinikum Olgahospital, (peds), CIC 701, S. Bielack, E. Koscielniak (5 (5) 0/5)
Stuttgart, Klinikum Stuttgart, Katharinenhospital, (ads), CIC 143, G. Illerhaus (58 (60) 8/50)
Tübingen, Universitätsklinikum, (ads), CIC 223, L. Kanz, C.Faul (116 (136) 57/59)
Tübingen, Universitätsklinikum, (peds), CIC 535, R. Handgretinger,P.Lang (36 (41) 24/12)
Ulm, Universitätsklinikum, (ads), CIC 204, D. Bunjes, H. Döhner (163 (186) 86/77)
Ulm, Kinderklinik Universitätsklinikum, (peds), CIC 204, A.Schulz (24 (32) 17/7)
Wiesbaden, Dr. Horst Schmidt Klinikum, (ads), CIC 586, N. Frickhofen, A. Brecht (29 (33) 0/29)
Winnenden, Rems-Murr Klinikum, (ads), CIC 180, M. Schaich (11 (20) 0/11)
Würzburg, Universitätsklinikum, (peds), CIC 196, P. Schlegel (14 (20) 7/7)
Würzburg, Universitätsklinikum, (ads), CIC 712, H.Einsele (191 (228) 70/121)

Greece: (12 teams: 330 (361) 193/137)
Alexandroupolis, Thrace University Med. School, (ads), I. Kotsianidis (0 (0) 0/0)
Athens, Attikon University General Hospital, (ads), CIC 604, P. Tsirigotis (41 (41) 34/7)
Athens, Hellenic Cancer Institute St. Savvas, (ads), CIC 751, A.Pouli, J. Filis (32 (37) 0/32)
Athens, Aghia Sophia Childrens Hospital, (peds), CIC 752, V. Kitra-Roussos (55 (65) 41/14)
Athens, Evanghelismos Hospital, (ads), CIC 622, D.Karakasis (55 (59) 36/19)
Athens, Diagnostic & Therapeutic Center 'Hygeia', (ads), CIC 643, G. Karianakis (11 (11) 0/11)
Athens, Athens Medical Center, (ads), A. Pigaditou (7 (7) 0/7)
Athens, G. Gennimatas Hospital, (ads), T.Marinakis, G. Gkortzolidis (4 (4) 0/4)
Athens, Laikon General Hospital, ads, CIC 438, J. Meletis, M. Angelopoulou (no report)
Crete, University Hospital, ads, H. Papadaki, C. Kalpadaki (no report)
Heraklion, Crete, University Hospital Heraklion, (peds), CIC 10788, E. Stiakaki (1 (1) 0/1)
Piraeus, Metaxa Cancer Hospital, (ads), CIC 937, C. Kosmas, E.Lianos, E. Fergadis (4 (8) 0/4)
Patras, University Hospital of Patras, (ads), CIC 281, A. Spyridonidis, M. Liga (31 (36) 25/6)
Thessaloniki, The George Papanicolaou General Hospital, (ads, peds), CIC 561, A. Anagnostopoulos (89 (92) 57/32)

Hungary: (5 teams: 375 (387) 111/264)
Budapest, Dél-pesti Centrumkórház, National Institute of Hematology, (ads), CIC 556, P.Remenyi, L. Gopcsa (179 (180) 70/109)
Budapest, Central Hospital of Southern Pest, (peds), CIC 824, G. Kriván (41 (46) 22/19)
Debrecen, University of Debrecen, (ads), CIC 648, A. Illes, L. Gergely (105 (110) 16/89)
Miskolc, GYEK, Child Health Centre, (peds), CIC 599, R. Simon, A.Kelemen (5 (6) 3/2)
Pécs, University of Pécs, (ads), CIC 682, A.Szomor (45 (45) 0/45)

Iceland: (1 team: no report)
Reykjavik, National University Hospital, (ads), CIC 605, S. Reykdal (no report)

Iran: (5 teams: 738 (753) 354/384)
Shiraz, Shiraz University of Medical Sciences, Nemazee Hospital, (ads, peds), CIC 188, M. Ramzi (155 (167) 54/101)
Teheran, Mofid Children Hospital, (peds), CIC 10190, B. Shahin Shamsian (40 (40) 34/6)
Teheran, Shariati Hospital, SCT Research Centre, (ads, peds), CIC 633, A. Mousavi (322 (322) 148/174)
Teheran, Childrens Medical Centre, (peds), CIC 856, Amir Ali Hamidieh (87 (90) 77/10)
Teheran, Taleghani Hospital Blood and Marrow Transplantation Center, (ads), CIC 916, M. Mehdizadeh, A. Hajifathali, M. Jahli (134 (134) 41/93)
Teheran, Mahak Children´s Cancer Hospital, peds, CIC 10224, A. Mehrvar (no report)
Urmia, Urmia Medical Sciences University, (ads), R. Asghari (no report)

Iraq: (1 team: 18 (20) 9/9)
Sulaimania Kurdistan, HIWA Cancer Hospital, (ads, peds), CIC 847, D. Othman, D. Hassan (18 (20) 9/9)

Ireland: (4 teams: 258 (289) 94/164)
Dublin, St. Vincent's Hospital, (ads), CIC 541, K. Fadalla (20 (20) 0/20)
Dublin, St.James Hospital, (ads, peds), CIC 257, K.Flynn, P. Browne, P.J. Hayden (153 (163) 82/71)
Dublin, Our Lady's Hospital of Sick Children, Crumlin, (peds), CIC 774, A. O'Marcaigh (20 (32) 12/8)
Galway, Galway University Hospitals, (ads), CIC 408, A. Hayat (65 (74) 0/65)

Israel: (10 teams: 681 (731) 349/332)
Beer Sheba, Soroka University Medical Center, (ads), CIC 481, I. Levy (30 (30) 1/29)
Haifa, Rambam Medical Center, (ads, peds), CIC 345, T. Zuckerman (142 (154) 76/66)
Jerusalem, Hadassah University Hospital, (ads, peds), CIC 258, P. Stepensky (54 (61) 31/23)*
Petach-Tikva, Childrens Medical Center, (peds), CIC 755, J. Stein (32 (40) 22/10)
Petach-Tikva, Beilinson Hospital, (ads), CIC 409, M. Yeshurun (86 (89) 44/42)
Revohot, Kaplan Hospital, (ads), CIC 327, L. Shvidel (5 (6) 0/5)
Tel Hashomer, Sheba Medical Center, (ads), CIC 754, A. Nagler, A. Shimoni (167 (175) 99/68)
Tel Aviv, Dana-Dwek Children's Hospital, Sourasky Medical Centre, (peds), CIC 670, R. Elhasid (16 (23) 7/9)
Tel Aviv, Tel Aviv Sourasky Medical Center, (ads), CIC 161, R. Ram (113 (113) 39/74)
Tel Hashomer, Chaim Sheba Medical Cebter, (peds), CIC 572, A. Toren (36 (40) 30/6)

Italy: (92 teams: 4,662 (5,416) 1,825/2,837)
Alessandria, S.S. Antonio e Biagio e C. Arrigo, (ads), CIC 825, M. Ladetto, M. Corsetti, F. Salvi, S. Tamiazzo (59 (68) 35/24)
Ancona, Azienda Ospedale Riuniti di Ancona, (ads), CIC 788, A. Olivieri (52 (59) 27/25)
Ancona, Azienda Ospedale Salesi Riuniti, (peds), P. Pierani, I. Carloni (3 (3) 0/3)
Ascoli Piceno, Mazzoni Hospital, (ads), CIC 119, P. Galieni (35 (46) 14/21)
Aviano, CRO IRCCS Aviano, (ads), CIC 162, M. Michieli, M. Rupolo, M. Mazzucato (36 (41) 0/36)
Avellino, A.O.S. Guiseppe Moscati, (ads), CIC 789, A. Risitano (41 (54) 11/30)
Bari, IRCCS Istituto Tumori "Giovanni Paolo II", (ads), CIC 934, A. Guarini (22 (22) 0/22)
Bari, Universitá degli Studi di Bari, (ads, peds), CIC 649, G. Specchia, P. Carluccio (44 (50) 25/19)
Barletta, Hospital of Barletta, (ads), CIC 555, G. Tarantini (15 (21) 0/15)
Bergamo, ASST Papa Giovanni XXIII, (ads), CIC 658, A. Rambaldi (111 (127) 53/58)
Bologna, Instituto Ortopedico Rizzoli, (ads, peds), CIC 453, A. Paioli (5 (5) 0/5)
Bologna, San Orsola-Malpighi Hospital, (ads), CIC 240, F. Bonifazi (118 (129) 41/77)
Bolzano, Ospedale San Maurizio, (ads), CIC 299, M. Casini, I. Cavattoni (70 (80) 32/38)
Bologna, Policlinico S. Orsola-Malpighi, (peds), CIC 790, A. Pession, A. Prete (16 (16) 8/8)
Brescia, Azienda Spedali Civili, (ads), CIC 288, G. Rossi, A. Re (110 (135) 0/110)
Brescia, Ospedale dei Bambini Spedali Civili, (peds), CIC 741, F. Porta (23 (23) 19/4)
Brindisi, Perrino Hospital, (ads), CIC 920, D. Pastore (28 (31) 15/13)
Brescia, Azienda Ospedaliera Spedali Civili Di Bresia, (ads), CIC 141, D. Russo (47 (47) 47/0)
Busto Arsizio, Ospedale di Circolo di Busto Arsizio, (ads), CIC 927, M. Bregni (11 (18) 0/11)
Cagliari, Ospedale per le Microcitemie, (peds), CIC 8112, M. Orofino (9 (12) 4/5)
Catania, Ospedale Ferrarotto, University of Catania, (ads, peds), CIC 792, G. Milone, G. Moschetti, L. Lo Nigro (38 (38) 23/15)
Cagliari, Binagh Hospital, Armando Businco Centre, (ads), CIC 8111, G. La Nasa, E Vacca (61 (68) 29/32)
Civitanove Marche, Ospedale di Civitanova Marche, (ads), CIC 419, R. Centurioni, M. Mirabile (9 (11) 0/9)
Como, Valduce Hospital, (ads), CIC 473, M.Turrini, F. Alberio, V. Saccà (15 (20) 0/15)
Cremona, U.O. Ematologia CTMO, (ads), A, Molteni, P. Spedini (7 (7) 0/7)
Cuneo, Azienda Ospedale "S. Croce e Carle", (ads), CIC 606, N.Mordini (34 (37) 17/17)
Ferrara, University of Ferrara, (ads), CIC 330, A. Cuneo (11 (16) 0/11)
Florence, Azienda Ospedaliera Universitaria di Careggi, (ads, peds), CIC 304, R. Saccardi, F. Bambi (125 (143) 56/69)
Foggia, Azienda Ospedaliero Universitaria, (ads), CIC 414, S. Capalbo, G. Spinosa (8 (9) 0/8)
Genova, Ospedale San Martino, (ads), CIC 217, E. Angelucci (118 (127) 58/60)
Genova, Istituto Giannina Gaslini, (peds), CIC 274, M. Faraci (34 (42) 15/19)

Latina, Ospedale Santa Maria Goretti, (ads), CIC 379, E. Ortu la Barbera (24 (36) 0/24)
Lecce, Opsedale Vito Fazzi de Lecce, (ads), CIC 868, N. Di Renzo (29 (37) 14/15)
Milan, Istituto Scientifico H.S. Raffaele, (ads, peds), CIC 813, F. Ciceri, M. Marcatti (136 (154) 86/50)
Milan, University of Milan IRCCS, (ads), CIC 265, G. Saporiti, S. Girelli (52 (64) 19/33)
Milan, Ist. Nazionale Tumori di Milano, (ads, peds), CIC 616, P. Corradini (70 (76) 16/54)
Milan, Ospedale di Niguarda, (ads), CIC 294, G. Grillo, C. Vigano (88 (96) 36/52)*
Milan, Instituto Europeo di Oncologia, (ads), CIC 331, R. Pastano. (38 (42) 17/21)
Milan, Istituto Clinico Humanitas IRCCS, (ads), CIC 354, L. Castagna, B. Sarina (104 (110) 46/58)
Milan, Azienda Socio Sanitaria Territoriale Fatebenefratelli Saccco, (ads), C. Bianchi. M. Bruno Vente (0 (0) 0/0)
Monza, Ospedale San Gerardo, Universita Di Milano-Bicocca, (ads), CIC 544, P. Pioltelli, M. Parma (72 (87) 39/33)
Modena, University of Modena, (ads, peds), CIC 543, F. Narni, P. Bresciani, G. Palazzi (46 (50) 13/33)
Monza, Ospedale San Gerardo, (peds), CIC 279, A. Bondi, A. Rovelli (36 (39) 35/1)
Napels, AORN Cardarelli Hospital, (ads), CIC 607, A. Picardi, M. Celentano, M. Pedata (70 (83) 28/42)
Napels, National Cancer Institute IRCCS, (ads), CIC 839, G. Marcacci, A. Pinto (44 (65) 0/44)
Napels, Hospital Pausilipon, (peds), CIC 341, V. Poggi, M. Ripaldi (23 (25) 14/9)
Napels, Frederico ll University, (ads), CIC 766, F. Pane, G. Battipaglia (36 (36) 7/29)
Pagani, Hospital A. Tortora, (ads), CIC 191, C. Califano (12 (13) 0/12)
Novara, Ospedale Maggiore della Carita, (ads), CIC 867, G. Gaidano, L. Nassi (37 (53) 0/37)
Nuoro, Ospedale San Francesco, (ads), CIC 793, A. Palmas, A. Uras (18 (18) 0/18)
Pavia, IRCCS Policlinico San Matteo, (ads, peds), CIC 562, P. Pedrazzoli (15 (39) 0/15)
Padova, Istituto Oncologia Veneto IOV-IRCCS, (ads), D.Marino (9 (11) 0/9)
Pavia, IRCCS Policlinico S. Matteo, (ads), CIC 286, P. Bernasconi, E.P. Alessandrino (79 (92) 31/48)
Palermo, Ospedale die Bambini, (peds), CIC 109, O. Ziino (4 (4) 1/3)
Pavia, Policlinico IRCCS St. Matteo, (peds), CIC 557, M.Zecca, L. Kelly (34 (34) 29/5)
Palermo, A.O.R. Villa Sofia Cervello, (ads), CIC 392, R. Scimè (49 (57) 28/21)
Padova, Clinica di Oncoematologia Pediatrica, (peds), CIC 285, E.Calore, A. Biff, C.Mainardi (27 (27) 18/9)
Palermo, Ospedale 'La Maddalena', (ads), CIC 692, M. Musso, F. Porretto, A. Crescinanno (128 (145) 31/97)
Palermo, ARNAS Civico Di Cristina, (ads), CIC 157, O. Ziino (12 (12) 0/12)
Parma, University of Palma, (ads), CIC 245, D. Vallisa, L. Prezioso (46 (51) 20/26)
Padova, Padua University Hospital, (ads), CIC 853, L. Trentin (58 (74) 0/58)
Pescara, Ospedale Civile, (ads, peds), CIC 248, S. Santarone, P. Di Bartolomeo (93 (95) 27/66)
Pesaro, AORMN Hospital, (ads), CIC 529, G.Visani (41 (45) 16/25)
Perugia, Ospedale Santa Maria della Misericordia, (ads, peds), CIC 794, A. Velardi, A. Carotti (84 (106) 27/57)
Piacenza, Hospital Guglielmo da Saliceto, (ads), CIC 163, D.Vallisa (39 (44) 16/23)
Pisa, University of Pisa, (ads, peds), CIC 795, M. Petrini, G. Casazza, M.Pelosini (73 (77) 33/40)
Potenza, San Carlo Hospital, (ads), CIC 861, M. Pizzuti, M. Cimminiello (15 (19) 1/14)
Ravenna, Romagna Metropolitan Transplant Network, (ads), CIC 306, F. Lanza (91 (147) 0/91)
Reggio Emilia, Arcispedale S. Maria Nuova, (ads), CIC 660, F. Merli, L. Facchini (32 (40) 7/25)
Reggio di Calabria, Grande Ospedale Metropolitano, Bianchi Melacrino Morelli, (ads, peds), CIC 587, G. Messina, M. Martino (98 (135) 31/67)
Rionero in Vulture, IRCCS Referral Cancer Center of Basilicata, (ads), CIC 185, Dr. Pietrantuono (8 (10) 0/8)
Rome, Rome Transplant Network, (ads), CIC 756, W. Arcese, P. De Fabritiis (165 (181) 56/109)
Rome, Università " La Sapienza", (ads, peds), CIC 232, R. Foa, A.P. Lori, S. Capria (80 (90) 37/43)
Rome, Università Cattolica S. Cuore, (ads), CIC 307, S. Sica, P. Chiusolo, A. Bacigalupo (113 (144) 66/47)
Rome, IRRCS Ospedale Bambino Gesu, (peds), CIC 796, F. Locatelli, F. Galaverna (144 (171) 110/34)
Rome, Ospedale S. Camillo, (ads), CIC 287, L. Rigacci (19 (19) 11/8)
Salerno, AOU San Giovanni di Dio e Ruggi D'Aragona Hospital, (ads), CIC 928, C. Selleri, B. Serio (20 (21) 10/10)
Sassari, Universita Di Sassari, (ads), CIC 870, F. Dore, L. Podda (12 (14) 0/12)
San Giovanni Rotondo, Hospital Casa Sollievo Sofferenza, (ads), CIC 526, AM. Carella (64 (72) 34/30)
Siena, Azienda Ospedaliera Universitaria Senese, (ads), CIC 321, G. Marotta, M. Tozzi (35 (35) 11/24)
Taranto, Institute of Haematologie, Ospedale Nord, (ads), CIC 332, P. Mazza, G. Palazzo (42 (43) 10/32)

Torino, A.O.U Citta della Salute e della Scienza di Torino, (ads), CIC 231, B. Bennedetto (142 (177) 56/86)
Torino, University Hospitals Torino, (ads, peds), CIC 305, M.Berger, F. Fagioli, F. Carnevale, D,Cilloni, A.Cignetti (95 (110) 46/49)
Trieste, Istituto per l'Infanzia, IRCCS Burlo Garofolo, (peds), CIC 525, N. Maximova, M. Rabusin (15 (15) 10/5)
Treviso, Presidio Ospedaliero Treviso, (ads), CIC 415, F. Gherlinzoni (47 (70) 0/47)
Trieste, Azienda Sanitaria Universitaria Integrata di Treste, (ads), CIC 982, F. Zaja, G. Desabbata (22 (23) 0/22)
Tricase (Lecce), Hospital C. Panico, (ads), CIC 652, V. Pavone (50 (50) 17/33)
Udine, Azienda Ospedaliero Universitaria di Udine, (ads), CIC 705, A. Sperotto, R. Fanin (96 (102) 63/33)
Varese, Ospedale di Circolo e Fondazione Macchi, (ads), CIC 878, A. Ferrario. B. Bianchi (29 (30) 0/29)
Venice, Ospedale dell'Angelo, (ads), CIC 502, R. Bassan, M. Vespignani (37 (40) 14/23)
Verona, Policlinico G. B. Rossi, (ads, peds), CIC 623, F. Benedetti, S. Cesaro (103 (105) 43/60)
Vicenza, Ospedale S. Bortolo, (ads), CIC 797, C. Borghero, M. Ruggeri (47 (51) 16/31)

Jordan: (1 team: 212 (213) 97/115)
Amman, King Hussein Cancer Centre, (ads, peds), CIC 580, A. Tbakhi (212 (213) 97/115)
Amman, Istishari Hospital, (ads), CIC 487, A. Ahmed Hussein (no report)

Kazakhstan: (1 team: 59 (59) 29/30)
Astana, National Research Center for Oncology and Transplantology, (ads), V. Kemaikin (59 (59) 29/30)

Latvia: (1 team: 31 (33) 1/30)
Riga, Clinic Linezers, (ads), S. Lejiniece, I. Trociukas (31 (33) 1/30)

Lebanon: (2 teams: 213 (216) 100/113)
Beirut, American University of Beirut, (ads, peds), CIC 369, A. Bazarbachi (104 (107) 47/57)
Bsalim, Middle East University Hospital, (ads, peds), CIC 477, A. Ibrahim (109 (109) 53/56)

Lithuania: (3 teams: 164 (197) 54/110)
Kaunas, University of Health Sciences Kauno Klinikos, (ads), CIC 942, R. Gerbutavicius (26 (26) 1/25)
Vilnius, University Childrens Hospital, (peds), CIC 508, J. Rascon (11 (12) 8/3)
Vilnius, Santariskiu Klinikos, (ads), CIC 644, L. Griskevicius, I. Trociukas (127 (159) 45/82)

Luxembourg: (1 team: 26 (26) 0/26)
Luxembourg, Center Hospitalier, (ads), S. De Wilde (26 (26) 0/26)

Macedonia: (1 team: 40 (40) 13/27)
Skopje, University Clinic for Haematology, (ads, peds), CIC 381, B. Georgievski (40 (40) 13/27)

The Netherlands: (14 teams: 1,506 (1,660) 613/893)
Amsterdam, Academic Med Centre, (ads), CIC 247, J. Zsivos, E. Nur (90 (99) 38/52)
Amsterdam, VU University Medical Center, (ads), CIC 588, E. Meijer, G.J. Ossenkoppele (193 (231) 73/120)
Amsterdam, Antoni Van Leeuwenhoek Hospital, (ads), CIC 976, S. C. Linn (17 (34) 0/17)
Enschede, Medisch Spectrum Twente, (ads), CIC 360, M.R. Schaafsma, MC. Legdeur (29 (29) 0/29)
Groningen, University Medical Centre UMCG, (ads), CIC 546, M.R. De Groot, G. Choi (167 (167) 81/86)
The Hague, Haga Hospital Leyenburg, (ads), CIC 547, M.R. Schipperus, S. Kersting (33 (38) 0/33)
Leiden, University Hospital, (ads, peds), CIC 203, J.H. Veelken (152 (160) 101/51)
Maastricht, University Hospital, (ads), CIC 565, G. Van Gorkom (133 (147) 43/90)
Nieuwegein, St. Antonius Hospital, (ads), CIC 200, H.K. Koene, O. de Weerdt (39 (41) 0/39)
Nijmegen, University Hospital, (ads), CIC 237, N. Schapp, T. De Witte (111 (125) 48/63)
Rotterdam, Erasmas MC Cancer Institute, (ads), CIC 246, J.J. Cornelissen, (259 (278) 108/151)
Utrecht, Princess Maxima Centre for Ped Oncology, (peds), CIC 352, M. Bierings (86 (101) 48/38)
Utrecht, University Medical Centre UMCU, (ads), CIC 239, E. Petersen (158 (166) 73/85)
Zwolle, Isala Klinieken, (ads), CIC 548, G.L. van Sluis (39 (44) 0/39)

Nigeria: (1 team: 1 (1) 0/1)

Benin, University Hospital Benin City, (ads), N. Bazuaye (1 (1) 0/1)

Norway: (5 teams: 388 (422) 158/230)
Bergen, Haukelands Sjukhus, (ads), CIC 197, A. Ahmed (41 (44) 12/29)*
Oslo, Oslo University Hospital, (ads, peds), CIC 235, T. Gedde-Dahl, J. Büchner (257 (277) 146/111)
Oslo, The Norwegian Radium Hospital, (ads, peds), CIC 782, G. Lauritzen, S. Kvaloy (41 (44) 0/41)
Tromso, University Hospital North Norway, (ads), A. Vik, G. Knutsen (16 (20) 0/16)
Trondheim, St. Olavs Hospital, (ads), O. Hjertner (33 (37) 0/33)

Poland: (18 teams: 1,395 (1,507) 588/807)
Bydgoszcz, Nicolaus Copernicus University, (peds), CIC 764, J. Styczynski, R. Debski (31 (35) 21/10)
Cracow, University Children's Hospital JUMC, (peds), CIC 507, J. Gozdzik (26 (26) 15/11)
Cracow, Jagiellonian University CMUJ, (ads), CIC 553, A. Skotnicki (78 (95) 27/51)
Gdansk, Medical University, (ads), CIC 799, M. Bieniaszewska (131 (131) 57/74)
Gliwice, Maria Curie Memorial Cancer Centre, (ads), CIC 428, S. Giebel (123 (137) 47/76)
Katowice, Silesian Medical Academy, (ads), CIC 677, G. Helbig (302 (312) 123/179)
Lodz, Medical University of Lodz, (ads), CIC 171, T. Robak (53 (54) 3/50)
Lublin, University Medical School, (ads), CIC 695, T. Gromek, M. Wach, A. Walter-Croneck, W. Legiec (39 (53) 7/32)*
Lublin, Childrens University Hospital, (peds), CIC 678, K. Drabko, J. Kowalczyk (15 (17) 12/3)
Poznan, Poznan University of Medical Sciences, (ads), CIC 730, L. Gil (118 (119) 63/55)
Poznan, University of Medical Sciences, Pediatric Hematology, (peds), CIC 641, J. Wachowiak (14 (15) 9/5)
Warsaw, Institue of Haematology and Blood Transfusion, (ads), CIC 693, K.Halaburda, B.Nasilowska, A.Tomaszewska (102 (114) 58/44)
Warsaw, Military Institute of Health Services, (ads), CIC 816, P. Rzepecki, K. Sulek (43 (50) 11/32)
Warsaw, Maria Sklodowska Curie National Research Institute, (ads), CIC 800, J. Walewski (65 (74) 0/65)
Warsaw, Central Clinical Hospital, (ads), CIC 954, G. Basak, P. Rusicka, W. Wiktor-Jedrzejczak, P. Boguradzki (56 (64) 25/31)
Wroclaw, Cape of Hope Medical University, (peds), CIC 817, A. Chybicka, K. Kalwak, J. Owoc-Lempach (54 (54) 46/8)
Wroclaw, University Hospital SPSK 1, (ads), CIC 699, T. Wrobel (124 (128) 54/70)
Wroclaw, Lower Silesian Center, (ads), CIC 538, A. Lange (21 (29) 10/11)

Portugal: (5 teams: 410 (453) 118/292)
Coimbra, University Hospital, (ads), CIC 905, C.Geraldes, A. Do Cén Teixeira, L. Ribeiro (38 (49) 0/38)
Lisbon, Instituto Portugues de Oncologia, (ads, peds), CIC 300, M. Nuno (87 (90) 36/51)
Lisbon, Hospital de Santa Maria, (ads, peds), CIC 636, F. Costa, F. Forjaz de Lacerda (76 (79) 26/50)
Lisbon, H. St. Antonio dos Capuchos, (ads), CIC 826, A. Botelho de Sousa (no report)
Porto, Instituto Portugues de Oncologia, (ads, peds), CIC 291, A. Campos (128 (150) 48/80)
Porto, Hospital St. Joao, (ads), CIC 329, J. E. Guimaraes, F. Trigo (81 (85) 8/73)

Romania: (5 teams: 156 (163) 59/97)
Bucharest, Coltea Clinical Hospital, (ads), CIC 912, A. Colita, A. Lupu, C. Ghimici (14 (16) 2/12)
Bucharest, Fundeni Clinical Centre, (peds), CIC 935, A. Colita, L. Dumitrache (14 (14) 11/3)
Bucharest, Fundeni Clinical Institute, (ads), CIC 427, A. Tanase (95 (99) 44/51)
Targu-Mures, Sectia Clinica de Hematologie si, (ads), CIC 178, I. Benedek (21 (22) 0/21)*
Timisoara, Emergency Hospital Louis Turcanu, (ads, peds), CIC 174, M. Serban, C. Jinca (12 (12) 2/10)

Russia: (13 teams: 1,260 (1,419) 588/672)
Ekaterinburg, Regional Hospital No. 1, (ads), T.S. Konstantinova, V.A. Shalaev (47 (47) 19/28)
Ekaterinburg, Royal Children's Hospital, (peds), CIC 884, L. Fechina (32 (35) 27/5)
Moscow, Burnasyan Fed. Med. Biophysical Centre, (ads), A. Davtyan, A.E. Baranov (23 (25) 0/23)
Moscow, Cancer Research Center N. N. Blokhin, (ads), G. Petrova, K. Kirgizov (46 (47) 0/46)
Moscow, N.N. Blokhin National Medical Research Center, (peds), CIC 893, K. Kirgizov (45 (46) 4/41)
Moscow, Research Haematology Center of RAS, (ads), CIC 930, V.G. Savchenko (220 (246) 91/129)
Moscow, Main Military Clinical Hospital, (ads), O.A. Rukavitsyn, V. Pop (11 (11) 0/11)
Moscow, The Russian Children's Research Hospital, (peds), CIC 411, E. Skorobogatova (55 (60) 47/8)
Moscow, Central Clinical Hospital (CCHPA), (ads), S. Shamansky (7 (10) 0/7)

Moscow, National Pirogov Medical Centre, (ads), V. Melnichenko, N. Mochkin (192 (192) 5/187)
Moscow, Federal Research Center for Pediatric Hematology, (peds), CIC 694, A. Maschan, D. Balachov (177 (240) 149/28)
Novosibirsk, Institute of Clinical Immunolgy, (ads), CIC 376, V. Sergeevuicheva (no report)
Samara, Samara Kalinin Regional Hospital, ads, V.A. Rossiev (no report)
St. Petersburg, Russian Scientific and Research Institute of Haematology, (ads), S. Voloshin (38 (44) 2/36)
St. Petersburg, First State Pavlov Medical University, (ads, peds), CIC 725, B.V. Afanasyev, L. Zubarovskaya (367 (416) 244/123)
St. Petersburg, Federal Centre V.A. Almazov, (ads), A. Zaritskey, D. Motorin (no report)
St. Petersburg, Research Institute of Oncology na N.N. Petrov, (ads, peds), CIC 845, S. Alekseev, I. Zyuzgin (no report)

Saudi Arabia: (5 teams: 484 (516) 340/144)
Dammam, King Fahad Specialist Hospital, (ads, peds), CIC 441, H. Al-Hashmi (no report)
Jeddah, King Faisal Hospital, (ads, peds), CIC 858, M. Bayoumi (53 (59) 37/16)
Riyadh, King Abdul Aziz Medical City, (ads, peds), CIC 444, M. Al Zahrani (129 (132) 93/36)
Riyadh, King Fahad Medical City, (ads, peds), CIC 159, M. Al-Harbi (44 (49) 23/21)
Riyadh, King Faisal Specialist Hospital, (ads, peds), CIC 397, M. Al Jurf (164 (171) 97/67)
Riyadh, King Faisal Specialist Hospital, (peds), CIC 981, M. Ayas, A. AlSeraihy (94 (105) 90/4)
Riyadh, Prince Sultan Military Medical City, (ads), CIC 818, S. Al Otaibi (no report)

Serbia: (4 teams: 132 (134) 42/90)
Belgrade, Clinical Center of Serbia, (ads), CIC 373, M. Todorovic Balint (73 (75) 22/51)
Belgrade, Military Medical Academy, (ads), CIC 582, D. Stamatovic (37 (37) 10/27)
Belgrade, Mother and Child Health Institute, (peds), CIC 358, D. Vujic (17 (17) 10/7)
Novi Sad, Clinical Center of Vojvodina, (ads), CIC 655, A. Savic (5 (5) 0/5)

Slovakia: (5 teams: 209 (234) 73/136)
Banská Bystrica, Roosevelt Hospital, (ads), CIC 333, I. Markuljak, E. Kralikova (12 (14) 0/12)
Bratislava, National Cancer Institute, (ads), CIC 368, A. Vranovsky (70 (76) 7/63)
Bratislava, University Hospital, (peds), CIC 684, J. Horàkovà, I. Bodova (23 (25) 16/7)
Bratislava, University Hospital, (ads), CIC 610, M.Mistrik (74 (89) 50/24)
Kosice, University Hospital, (ads), N. Stecova, T. Guman (30 (30) 0/30)

Slovenia: (1 team: 122 (145) 35/87)
Ljublijana, University Medical Centre, (ads, peds), CIC 640, M. Sever, V. Rajic (122 (145) 35/87)

South Africa: (9 teams: 297 (310) 121/176)
Bloemfontein, Free State University Hospital, (ads), J. Malherbe (2 (2) 0/2)
Cape Town, Constantiaberg Medical Clinic, (ads, peds), CIC 772, M. Du Toit (20 (20) 3/17)
Cape Town, Groote Schuur Hospital, (ads, peds), CIC 512, E. Verburgh (41 (41) 21/20)
Cape Town, Netcare Kuils River Hospital, (ads, peds), H. Koornhof (66 (68) 30/36)
Cape Town, Melomed Hospital Tokai, (ads, peds), S. Nahrwar (no report)
Cape Town, UCT Private Academic Hospital, ads, peds, CIC 512, N. Novitzky (no report)
Durban, Inkosi Albert Luthuli Hospital, (ads, peds), S. Parasnath (6 (6) 2/4)
Durban, Capital Haematology Hospital, (ads), J.P.Singh (27 (27) 1/26)
Durban, Netcare Umhlanga Hospital, (ads), N. Sewpersad (no report)
Johannesburg, Wits Donald Gordon Medical centre, (ads, peds), CIC 483, J. Thomson (18 (18) 8/10)
Johannesburg, Baragwanath Hospital, (ads), M. Patel, V. Philips (no report)
Pretoria, Netcare Pretoria East Hospital, (ads, peds), CIC 456, D. Brittain, A. McDonald (99 (110) 49/50)*
Randburg, Netcare Olivedale Hospital, (ads), CIC 889, K. Gunther. D. Brittain (18 (18) 7/11)

Spain: 69 teams: 2,994 (3,200) 1,227/1,767)
Alicante, Hospital Universitario Torrecardenas, (ads), CIC 486, M.J. Garcia Perez (4 (4) 0/4)
Alicante, Hospital General, (ads), P. Fernandez Albellan (28 (28) 0/28)
Barcelona, Hospital Sant Joan de Deu, (peds), CIC 668, I. Badell-Serra (24 (32) 14/10)
Barcelona, Santa Creu i San Pau, (peds), CIC 260, I. Badell Serra, M. Torrent (7 (8) 7/0)
Barcelona, Santa Creu i Sant Pau, (ads), CIC 260, J. Sierra, S. Brunet, A. Esquirol (84 (86) 43/41)

Barcelona, Institute Catala d'Oncologia, Hospital Duran i Reynals, (ads), CIC 759, A. Mussetti,, A. Sureda (104 (109) 50/54)
Barcelona, Hospital General Vall d'Hebron, (ads), CIC 584, D. Valcarcel (54 (57) 26/28)
Barcelona, Hospital Mutua de Terrassa, (ads), J. M. Marti Tutusaus (36 (39) 0/36)
Barcelona, Hospital Clinic, (ads), CIC 214, M. Rovira (88 (92) 44/44)
Barcelona, Hospital M. Infantil, Vall d'Hebron, (peds), CIC 422, C. Diaz de Heredia (24 (29) 19/5)
Barcelona, Hospital Germans Trias i Pujol, (ads, peds), CIC 613, J M. Ribera Santasusana (49 (51) 33/16)
Caceres, Hospital San Pedro de Alcantara, (ads), J. Prieto, JM. Bergua (39 (39) 0/39)
Cadiz, Hospital del SAS, (ads), S. Garzon Lopez (43 (47) 21/22)
Castellon de La Plana, Hospital General de Castellon, ads, CIC 844, R. Garcia-Boyero (20 (25) 0/25)**.
La Coruna, Complejo Hospitalario de A Coruna, (ads), CIC 361, J P. Torres Carrete, M R. Varela Gomez (45 (50) 19/26)
Cordoba, Hospital Reina Sofia, (ads, peds), CIC 238, C. Herrere Arroyo, C. Martin Calvo, V. Martin Palanco (58 (60) 30/28)
Barakaldo Vizcaya, Hospital de Cruces, (ads, peds), CIC 393, J. Garcia-Ruiz, J. Mateos-Mazon (74 (87) 0/74)
Galdakao, Hospital de Galdakao, (ads), J.Ojanguren, T.Carrascosa, K.Atutxa (13 (13) 0/13)
Girona, Institut Catala d'Oncologia, Josep Trueta, (ads), CIC 433, D. Gallardo (20 (20) 0/20)
Granada, Hospital Virgen de la Nieves, (ads), CIC 559, M. Jurado Chacon (76 (77) 33/43)
Jaen, Hospital Cuidad de Jaen, (ads), F. Almagro Torres (16 (17) 0/16)
La Laguna, Tenerife, University Hospital Canary Isles, (ads), M.T. Hernandez-Garcia, B. Soris S. Barbara (no report)
Las Palmas Canary Isles, Hospital Insular, (ads), J. Gonzalez-San Miguel (27 (28) 0/27)
Las Palmas Canary Isles, Hospital de Gran Canaria 'Dr. Negrin', (ads, peds), CIC 537, M.del Mar Perera, A.Suarez, H.Luzardo (53 (54) 30/23)
Lleida, Hospital Arnau de Vilanova, (ads), CIC 885, A. Garcia Guinon (15 (15) 0/15)
Leon, Hospital Universitario de Leon, (ads), CIC 426, N de Las Heras (20 (20) 0/20)
Logrono, Hospital San Pedro, La Rioja, (ads), CIC 917, M. Najera Irazu, M. Hermosilla (20 (20) 0/20)
Lugo, Hospital Lucus Augusti, (ads), J. Arias Sampedro (9 (10) 0/9)
Madrid, Hospital Universitario La Paz, (ads, peds), CIC 734, A. Perez-Martinez, R. De Paz (76 (76) 43/33)
Madrid, Hospital Univeristario Materno Infantil Gregorio Maranon, (peds), CIC 410, C. Belendez (16 (16) 15/1)
Madrid, Hospital Universitario San Carlos, (ads), M.Paz Martin, C. Benavente (11 (11) 0/11)
Madrid, Hospital General Universitario Gregorio Maranon, (ads, peds), CIC 819, J.L. Diez-Martin, P. Balsalobre (64 (72) 38/26)
Madrid, Hospital Universitario Quironsalud and Hospital Moncloa, (ads), JM. Fernadez-Ranada, A. Escudero, M. Chamarro (24 (24) 5/19)
Madrid, Hospital Universitario Puerta de Hierro, (ads), CIC 728, JR Cabrera Martin, G. Bravo (39 (42) 21/18)
Madrid, Hospital de la Princesa, (ads), CIC 236, A. Figuera, A. Alegre (42 (43) 26/16)
Madrid, Hospital Ramon y Cajal, (ads), CIC 615, J. Lopez-Jimémez (72 (72) 21/51)
Madrid, Hospital Principe Asturias, Alcala de Henares, (ads), Dr. Lopez Rubio, E. Magro Mazo (2 (2) 0/2)
Madrid, Hospital Doce de Octubre, (ads, peds), CIC 382, J. Martinez, M.L.Paciello (76 (76) 22/54)
Madrid, Hospital Universitario de Getafe, (ads), L.Garcia Alonson, F.Oña Compan, N.Somolinos, C.Monteserin (14 (14) 0/14)
Madrid, Hospital Niño Jesus, (peds), CIC 732, M.A. Diaz (47 (60) 38/9)
Malaga, Hospital Regional Malaga, (ads, peds), CIC 576, M-J. Pascual-Cason (132 (136) 71/61)
Malaga, Hospital Virgen de la Victoria, (ads), CIC 476, A. Rosell Mas (no report)
Madrid, Hospital Universitario Sanchinarro, (ads), J. Pérez de Oteyza (14 (14) 1/13)
Madrid, Hospital Universitario Sanitas La Zarzuela, (ads), CIC 779, R. De la Camara (0 (0) 0/0)
Madrid, Fundacion Jimenez Diaz, (ads), CIC 309, JL. Lopez-Lorenzo (59 (61) 24/35)
Murcia, Hospital Virgen de la Arrixaca, (ads, peds), CIC 323, JM. Moraleda, A. Sanchez-Salinas (45 (57) 22/23)
Murcia, Hospital General Universitario Morales Meseguer, (ads), CIC 735, I. Heras, V. Vicente-Garcia (49 (57) 18/31)
Madrid, Hospital Severo Ochoa, Leganés, (ads), P. Sanchez Godoy (7 (7) 0/7)
Orense, Com. Hospital Cristal-Pinor, (ads), J-L. Sastre-Moral (12 (12) 0/12)
Oviedo, Hospital Covadonga, Central Asturias, (ads, peds), CIC 642, S. Gonzalez-Müniz (84 (85) 30/54)

Palma de Mallorca, Hospital son Llatzer, (ads), CIC 110, J. Bargay-Lleonart (15 (16) 0/15)
Palma de Mallorca, Hospital Uni. Son Espases, (ads, peds), CIC 722, A. Sampol (43 (49) 16/27)
Pamplona, Hospital de Navarra, (ads), CIC 577, T. Zudaire (47 (48) 17/30)
Pamplona, Clinica Universitaria de Navarra, (ads, peds), CIC 737, J. Rifon (40 (42) 17/23)
Pontevedra, Hospital Montecelo, (ads), A-M. Dios Loureiro (13 (13) 0/13)
Santiago de Compostela, Hospital Clinico Universitario, (ads, peds), CIC 570, J.L. Bello Lopez (50 (54) 26/24)
Salamanca, Hospital Clinico, (ads, peds), CIC 727, D. Caballero (146 (152) 88/58)
Santander, Hospital Universitario Marqués de Valdecilla, (ads, peds), CIC 242, M. Colorado Araujo (67 (74) 38/29)
San Sebastian, Hospital Universitario Donostia, (ads), C. Vallejo Llamas,JJ Ferreiro Martinez (82 (96) 48/34)
Sevilla, Hospital Universitario Virgen del Rocio, (ads, peds), CIC 769, J. A. Pérez-Simón (144 (158) 55/89)
Tarragona, Hospital Joan XXIII de Tarragona, (ads), C. Talarn Forcadell (25 (26) 0/25)
Tenerife Canary Isles, Hospital N. S. De la Candelaria, (ads, peds), J. Garcia-Talavera, J. Breña, P. Rios Rull (27 (28) 0/27)
Valencia, Hospital Universitario La Fe, (peds), CIC 653, J.M. Fernandez Navarro (27 (36) 21/6)
Valencia, Hospital Clinico de Valencia, (ads, peds), CIC 282, C. Solano (69 (78) 31/38)
Valladolid, Hospital Rio Hortega, (ads), CIC 611, J. Garcia Frade (31 (31) 10/21)
Valencia, Instituto Valenciano de Oncologia, (ads), A. Avaria, C. Salazar (0 (0) 0/0)
Valencia, Hospital Arnau de Vilanova de Valencia, (ads), A. Lopez Martinez (11 (12) 0/11)
Valencia, Hospital Doctor Peset, (ads), P. Ribas-Garcia, A. Garcia Feria (22 (30) 0/22)
Valencia, Hospital Universitario La Fe, (ads, peds), CIC 663, J. Sanz, G.F.Sanz (130 (132) 72/58)
Vigo, CHUVI Hospital Alvaro Cunqueiro, (ads), CIC 421, C. Albo Lopez (36 (40) 17/19)
Zaragoza, Hospital Miguel Servet, (ads), P. Delgado (35 (37) 7/28)
Zaragoza, Clinico Universitario Lozano Blesa, (ads), L. Palomera Bernal (19 (19) 0/19)

Sweden: (7 teams: 696 (786) 263/433)
Goteborg, CHECT Sahlgrenska University Hospital, (ads, peds), CIC 289, J. Johansson, K. Mellgren (129 (153) 46/83)
Stockholm, Karolinska University Hospital, (ads, peds), CIC 212, S. Mielke, J. Winiarski, P. Ljungman (191 (197) 86/105)
Linköping, University Hospital, (ads), CIC 740, J. Cammenga (67 (76) 22/45)
Lund, University Hospital, (ads, peds), CIC 283, S. Lenhoff, J. Toporski (129 (145) 56/73)
Örebro, Medical Center Hospital, (ads), CIC 738, P. Kozlowski (29 (35) 0/29)
Umea, Umea University Hospital, (ads), CIC 731, C. Isaksson (48 (62) 16/32)
Uppsala, University Hospital, (ads, peds), CIC 266, K. Carlson, N. Jackmann (103 (118) 37/66)

Switzerland: (10 teams: 683 (809) 265/418)
Aarau, Kantonsspital Aarau, (ads, peds), CIC 316, M. Bargetzi, S. Gerull (25 (38) 0/25)
Basel, Universitätsspital Basel, (ads, peds), CIC 202, J. Passweg, D. Heim, J.Halter (128 (148) 86/42)
Bellinzona, Ospedale San Giovanni, (ads), CIC 829, L. Wannesson (17 (18) 0/17)
Bern, Inselspital, (ads, peds), CIC 221, T. Pabst, J. Rössler, G. Baerlocher (126 (163) 0/126)
St. Gallen, Kantonsspital, (ads), CIC 324, F. Hitz (41 (47) 0/41)
Geneva, Hôpital Cantonal Universitaire, (ads, peds), CIC 261, Y. Chalandon, M. Ansari (71 (75) 71/0)
Lausanne, CHUV, (ads), CIC 820, M. Duchosal (74 (86) 0/74)
Zurich, Hospital Hirslanden, (ads), CIC 638, Ch. Renner (26 (29) 0/26)
Zurich, University Hospital, (ads), CIC 208, U. Schanz, G. Nair, A. Müller (147 (172) 81/66)
Zurich, Universitäts Kinderklinik, (peds), CIC 334, T. Güngör (28 (33) 27/1)

Syria: (1 team: no report)
Damascus, Tishreen Hospital, (ads), CIC 10853, De. S. Elias (no report)

Tunisia: (1 team: 76 (81) 36/40)
Tunis, National BMT Centre, (ads, peds), CIC 183, B. Othman Tarck (76 (81) 36/40)

Turkey: (75 teams: 4,373 (4,560) 2,157/2,216)
Adana, Adana Acibadem Hospital, (peds), CIC 454, A. Antmen (60 (60) 59/1)
Adana, Baskent University Adana, (ads), CIC 589, H. Ozdogu, C. Boga, S. Asma (90 (95) 39/51)

Ankara, Yildirim Bayazit Training and Research Hospital, (ads), M. Albayrak (9 (9) 0/9)
Ankara, Dr. A. Yurtaslan Oncology Training and Research Hospital, (ads), F. Altuntas, M. Sinan Dal (97 (97) 50/47)
Ankara, Memorial Hospital Ankara, (ads), F. Avcu (39 (40) 21/18)
Antalya, Medical Park Antalya Hospital, Lara, (peds), CIC 911, A. Yesilipek (88 (98) 84/4)
Ankara, Ankara Bayindir Hospital, (ads), CIC 412, A.Ural (49 (50) 25/24)
Antalya, Antalya Education and Research Hospital, (ads), CIC 914, E. Kurtoglu, I. Nizam Özen (41 (41) 9/32)
Ankara, Gazi University Medical School, Besevler, (peds), CIC 182, U. Kocak (10 (10) 6/4)
Ankara, Gazi University Medical School, Besevler, (ads), CIC 169, Z.N. Ozkurt, Ö. Karacaoglu (57 (68) 32/25)
Ankara, Ankara Baskent Hospital, (ads), S. Zeynep Aki, E. Koca (15 (16) 5/10)
Antalya, Medstar Antalya Hospital, Cakirlar, (ads), CIC 864, I. Karadogan (93 (98) 26/67)
Ankara, Lösante Hospital, (peds), A.Emin Kurekci (27 (31) 27/0)
Ankara, Ankara University Faculty of Medicine, Dikimevi, (ads), CIC 617, P. Topguoglu, G. Gürmann, M. Kurt Yüksel (94 (108) 33/61)
Ankara, SBU Gülhane Training and Research Hospital, (ads), N. Karadurmus, O. Gürsel, R. Acar (76 (76) 0/76)
Ankara, Hacettepe University Medical School, Sihhiye, (ads), CIC 168, H. Goker (75 (75) 33/42)
Ankara, Hacettepe Ihsan Dogramaci Childrens Hospital, (peds), CIC 399, D. Uckan-Cetinkaya, B. Kuskonmaz (18 (18) 18/0)
Ankara, Children's State Hospital, (peds), CIC 436, B. Tunc (55 (55) 47/8)
Antalya, Akdeniz University School of Medicine, (ads), CIC 685, L. Undar (32 (34) 11/21)
Ankara, Ozel Koru Hospital, (ads), A. Ugur Bilgin (62 (62) 47/15)
Ankara, University of Ankara, Cebeci, (peds), CIC 620, T. Lleri, E. Unal (17 (21) 13/4)
Antalya, Akdeniz University Medical School, (peds), CIC 618, A. Kupesi (36 (39) 34/2)
Ankara, Private Medicana International Hospital, (ads), E. Soydan (140 (155) 67/73)
Aydin, Adnan Menderes University Medical Faculty, (ads), CIC 187, Z. Bolaman, I. Yavasoglu (51 (51) 10/41)
Adana, Balcali Hospital, Cukurova University, (ads), CIC 462, B. Güvenc (22 (23) 12/10)
Adana, Cukurova University Balcali Hospital, (peds), G. Inan (39 (42) 34/5)
Bursa, Uludag University School of Medicine, (peds), AM. Günes, M. Evim (12 (12) 8/4)
Bursa, Uludag University School of Medicine, (ads), V. Özkocaman (49 (49) 16/33)
Denizli, Pamukkale University Hospital, (ads), S. Kabukcu (35 (35) 0/35)
Diyarbakir, Dicle University Faculty, (ads), O. Ayyildiz (12 (12) 0/12)
Erzurum, Atatürk University, (ads), Y. Bilen (no report)
Eskisehir, Osmangazi University, (ads), E. Gündüz (33 (33) 5/28)
Gaziantep, Gaziantep University Medical School, (ads), CIC 402, M. Pehlivan (20 (23) 19/1)
Istanbul, Medical Park Göztepe, (ads), C. Adigüzel (116 (116) 52/64)
Istanbul, Koç University Hospital, (ads), CIC 943, O. Akay (57 (57) 10/47)
Istanbul, Florence Nightingale Sisli Hospital, (ads), CIC 994, M. Arat (91 (91) 44/47)
Istanbul, Yeditepe Universtity Hospital, (peds), BE. Del Castello (2 (2) 2/0)
Istanbul, Emsey Hospital, (ads), CIC 355, S. Omay, Y. Ünsal (57 (57) 35/22)
Istanbul, Bahcelievler Medical Park Hospital, (ads, peds), G. Sucak (88 (101) 56/32)
Istanbul, Istanbul Medipol University, (ads), CIC 445, D. Sargin (75 (75) 26/49)
Istanbul, Hisar Intercontinental Hospital, (ads), A. Timuragaoglu (49 (49) 21/28)
Istanbul, Cerrahpasa Medical Faculty, (ads), CIC 761, T. Soysal, M. Cem Ar, T. Elverdi (43 (43) 9/34)
Istanbul, Yeniyuzyil University, Gaziosmanpasa Hospital, (ads), CIC 475, H. Goksoy (112 (112) 56/56)
Istanbul, Istanbul Medipol University, (peds), CIC 446, Y. Yaman, S. Anak (41 (41) 22/19)
Istanbul, Yeditepe University Hospital, (ads), CIC 416, A. Özkan (41 (43) 19/22)
Istanbul, Medicana International Hospital, (ads), Y. Koc (32 (36) 16/16)
Istanbul, Medical Park Goztepe, (peds), G. Karasu, SC. Kilic (84 (98) 78/6)
Istanbul, Medical Park Hospitals, (ads), CIC 919, Y. Koc (39 (43) 23/16)
Istanbul, Acidadem Atakent Hospital, (ads), S. Sami Karti, A. Uzay (190 (201) 65/125)
Ankara, Liv Hospital, (ads), O. Nevruz (63 (63) 19/44)
Istanbul, Acibadem University Altunizade Hospital, (peds), CIC 457, G. Öztürk (46 (46) 42/4)
Istanbul, Acibadem University Altunizade Hospital, (ads), CIC 468, S. Ratip, E. Ovali (81 (81) 41/40)
Istanbul, Sisli Memorial Hospital, (ads), S. Izmir Güner (101 (101) 51/50)
Istanbul, University of Istanbul, (ads), CIC 760, I. Yonal-Hindilerden, M. Aktan (41 (45) 19/22)
Istanbul, Bahcelievler Memorial Hospital HSCTU Center, (ads), E. Tekgündüz (74 (74) 41/33)

Istanbul, Marmara University Hospital, (ads), CIC 714, T. Firatli-Tuglular, T. Toptas (25 (25) 10/15)
Istanbul, Medical Park Bahcelievler Hospital, (peds), CIC 4482, T. Fisgin, C. Bozkurt (73 (81) 65/8)
Istanbul, Yeniyuzyil University, Gaziosmanpasa Hospital, (peds), CIC 459, B. Malbora (79 (79) 73/6)
Istanbul, Sisli Memorial Hospital, peds, A. Tanyeli (no report)
Istanbul, Kolan International Hospital, ads, S. Dincer (no report)
Izmir, Ege University Medical Faculty, Bornova, (ads), CIC 628, F. Vural, G. Saydam, N. Soyer (68 (68) 26/42)
Izmir, Medicalpark Private Hospital, Karsiyaka, (ads), S.Cagirgan, Kahraman, C. Acarlar (94 (94) 46/48)
Izmir, Ege University Medical Faculty, Bornova, (peds), CIC 621, S. Kansoy (31 (33) 25/6)
Izmir, Kent Hospital, (ads), G. Kadiköylü (113 (113) 41/72)
Izmir, Tepecik Research and Educational Hospital, (peds), H. Öniz (1 (1) 1/0)
Izmir, Dokuz Eylul University, (ads, peds), CIC 688, G. H. Özsan. H. Ören (43 (43) 9/34)
Kayseri, Erciyes University Faculty of Medicine, (peds), CIC 913, M. Karakukcu (44 (55) 35/9)
Kayseri, Erciyes University Hospital, (ads), CIC 627, A. Unal, M. Cetin (103 (108) 46/57)
Kocaeli, Anadolu Medical Center Hospital, (ads), CIC 440, Z. Gülbas (288 (314) 130/158)
Kocaeli, Kocaeli University Hospital, (ads), P. Tarkun (31 (31) 0/31)
Konya, Necmettin Erbakan, Meram University Medical Hospital, (ads), Ö. Ceneli (30 (30) 0/30)
Kocaeli, Kocaeli University Hospital, (peds), E. Zengin (7 (7) 6/1)
Malatya, Inönu University Hospital, (peds), A. Akyay (11 (11) 9/2)
Manisa, Celal Bayer University, (ads), I. Aydogdu (14 (14) 1/13)
Malatya, Inonu University Targut Özal Medical Centre, (ads), M. Ali Erkurt, E. Kaya (179 (179) 61/118)
Samsun, Ondokuz Mayis University, (peds), C. Albayrak (23 (23) 14/9)
Atakum, Özel Sansung Medicalpark Hospital, (peds), CIC 881, H.E. Ozyurek, E. Sahin (6 (6) 6/0)
Trabzon, KTU Farabi Hospital, (ads), E. Nas, M. Sonmez (34 (34) 16/18)

Ukraine: (4 teams: 131 (145) 22/109)
Cherkasy, Regional Oncology Hospital, (ads), V. Paramonov (new in 2021)
Kiev, National Pediatric Specialized Hospital, OHMATDYT, (peds), CIC 10109, O. Lysytsia (26 (26) 22/4)
Kiev, National Cancer Centre, (ads), CIC 10832, K. Filonenko, E. Kushcheviy (21 (21) 0/21)
Kiev, Kiev BMT Center MNPE KBMTC, (ads, peds), E. Karamanesht, V. Khomenko, I. Korenkova (60 (69) 0/60)
Kiev, National Cancer Institute, (peds), S. Pavlyk (24 (29) 0/24)

United Kingdom: (49 teams: 3,169 (3,460) 1,375/1,794)
Aberdeen, The Royal Infirmary, (ads), CIC 344, D.J.Culligan (20 (21) 0/20)
Bath, Royal United Hospital, (ads), CIC 619, J. Crowe (18 (18) 0/18)
Belfast, Belfast City Hospital Trust, (ads), CIC 268, D. Finnegan (47 (49) 11/36)
Birmingham, The Birmingham Children's Hospital, (peds), CIC 781, S. Lawson (25 (30) 17/8)
Birmingham, Queen Elizabeth Hospital, (ads, peds), CIC 387, R. Malladi (158 (166) 89/69)
Birmingham, Heartlands Hospital, (ads), CIC 284, S. Paneesha (24 (28) 16/8)
Blackpool, Victoria Hospital, (ads), CIC 832, M.P. Macheta (28 (29) 0/28)
Bournemouth, Royal Bournemouth Hospital, (ads), CIC 765, R. Hall (20 (22) 0/20)
Bristol, Avon and Royal Hospital for Sick Children, (ads, peds), CIC 386, R. Protheroe, D. Marks, S. Robinson (120 (124) 84/36)
Cambridge, Addenbrooke's Hospital, (ads), CIC 566, C.Crawley, J Craig (112 (128) 57/55)
Cardiff/Swansea, University Hospital of Wales, (ads, peds), CIC 303, K.M.O. Wilson, P. Connor, W. Ingram (90 (97) 45/45)
Cheltenham, Cheltenham General Hospital, (ads), CIC 398, S. Chown (7 (8) 0/7)
Coventry, University Hospital Coventry & Warwickshire NHS Trust, (ads), CIC 322, F. Jones (15 (15) 0/15)
Dundee, Ninewells Hospital, (ads), CIC 719, D. Meiklejohn (6 (6) 0/6)
Dudley, Dudley NHS Trust, (ads), CIC 405, S. Fernandes (3 (3) 0/3)
Edinburgh, The Western General Hospital, (ads), CIC 228, A. J.M. Broom, F.Scott, P.Roddie (36 (41) 0/36)
Exeter, Royal Devon and Exeter Hospital, (ads), CIC 571, P. Kerr (14 (14) 0/14)
Glasgow, Royal Hospital for Children, (peds), CIC 707, B. Gibson (17 (20) 12/5)
Glasgow, Beatson, West of Scotland Cancer Centre, (ads, peds), CIC 244, I.G. McQuaker, A. Parker (123 (129) 63/60)
Leeds, Yorkshire Hospitals NHS Trust, (ads, peds), CIC 254, M. Gilleece, J.Ashcroft. R. Patmore (157 (170) 69/88)

Leicester, Royal Infirmary Hospital, (ads), CIC 713, M. Martin (50 (51) 15/35)
Liverpool, Alder Hay, (peds), CIC 773, M. Caswell (3 (6) 0/3)
Liverpool, Royal Liverpool University Hospital, (ads), CIC 501, A. Patel, R. Salim (56 (57) 14/42)
London, St Mary's Hospital, (peds), CIC 866, J de La Fuente (17 (18) 17/0)
London, Hammersmith Hospitals NHS Trust, (ads, peds), CIC 205, J.Apperley, E. Kanfer, D. Slade, R. Szydlo (103 (121) 49/54)
London, The London Clinic, (ads), CIC 263, M. Potter (38 (40) 8/30)
London, London Bridge Hospital, (ads), CIC 460, M. Kazmi (3 (3) 0/3)
London, St. George's Hospital, (ads), CIC 539, M. Koh, M. Klammer (39 (50) 18/21)
London, University College Hospital, (ads, peds), CIC 224, B. Carpenter (265 (294) 100/165)
London, King's College Hospital, (ads), CIC 763, G. Mufti, V. Potter (161 (178) 61/100)
London, Royal Marsden Hospital, (ads, peds), CIC 218, M. Potter (174 (191) 80/94)
London, St. Bartholomew's and the Royal London NHS Trust, (ads), CIC 768, J. Gribben, S. Montoto,J. Cavenagh, S. Agrawal (100 (111) 26/74)
London, Great Ormond Street Hospital, (peds), CIC 243, P. Veys (72 (80) 56/16)
Manchester, Christie NHS Trust Hospital, (ads, peds), CIC 780, A.Bloor (124 (129) 55/69)
Manchester, Central Manchester NHS Trust, (peds), CIC 521, R. F. Wynn (49 (57) 43/6)
Manchester, The Royal Infirmary, (ads), CIC 601, E. Tholouli (120 (120) 73/47)
Newcastle upon Tyne, Freeman Hospital, (ads, peds), CIC 276, M. Collins, M. Slatter (170 (185) 90/80)
Norwich, The Norfolk and Norwich University Hospital, (ads), CIC 391, M. Lawes (21 (22) 0/21)
Nottingham, Nottingham City Hospital, (ads, peds), CIC 717, J.L. Byrne (112 (121) 45/67)
Oxford, John Radcliffe Children's Hospital, (peds), CIC 603, G. Hall (3 (6) 0/3)
Oxford, Cancer and Haematology Centre, Churchill Hospital, (ads), CIC 255, A. Peniket,T.Littlewood,A.Brown,G.Collins (98 (98) 45/53)
Plymouth, University Hospitals Plymouth NHS Trust, (ads), CIC 823, H.M.Hunter (85 (91) 31/54)
Poole, Poole Hospital NHS Foundation Trust, (ads), CIC 458, J. Fergus (8 (8) 0/8)
Sheffield, Teaching Hospitals NHS Trust, Children's Hospital, (ads), CIC 778, J.Snowden (89 (97) 38/51)
Sheffield, Childrens NHS Foundation, (peds), CIC 933, K. Patrick (16 (19) 9/7)
Taunton, Taunton and Somerset NHS Foundation Trust, (ads), CIC 708, S. Bolam (9 (9) 0/9)
Southampton, University Hospital Southampton NHS Foundation Trust, (ads, peds), CIC 704, K. Orchard, D. Richardson (113 (147) 39/74)
Stoke-on-Trent, University Hospitals of North Midlands, (ads), CIC 394, S. Pillai, R. Chasty (20 (21) 0/20)
Swindon, Great Western Hospital, (ads), CIC 608, N. E. Blesing (11 (12) 0/11)

*EBMT database extract
**Late report: data not included in the analysis.

Europe total: 690 teams: (41,016 (45,364) 17,647/23,369)
